# Neuro-immune interactions in coronary microvascular disease: mechanisms and therapeutic prospect

**DOI:** 10.3389/fimmu.2025.1631083

**Published:** 2025-08-29

**Authors:** Zhao Ge, Guoyu Wu, Jingguo Ma, Silin Ren, Tongzuo Liu, Xudong Wu, Xianliang Wang

**Affiliations:** 1 First Teaching Hospital of Tianjin University of Traditional Chinese Medicine, National Clinical Research Center for Chinese Medicine Acupuncture and Moxibustion, Tianjin, China; 2 Graduate School, Tianjin University of Traditional Chinese Medicine, Tianjin, China

**Keywords:** neuroimmunology, central nervous system, brain, mucosal immunology, coronary microvascular dysfunction, autonomic nervous system, precision cardiovascular therapy

## Abstract

Coronary microvascular disease (CMVD) represents a widespread but frequently underdiagnosed cardiovascular condition marked by endothelial dysfunction and inflammatory microvascular remodeling. Mounting evidence underscores the pivotal involvement of neuro-immune interactions in CMVD pathogenesis, encompassing complex bidirectional communication among the autonomic nervous system, immune cells, and vascular endothelium. This review first examines the evolving paradigm of neuro-immune cardiovascular circuits and elucidates critical signaling pathways—including NF-κB, MAPK, mTOR, STAT3, and cAMP—that govern these interactions. Subsequently, to provide a clinical context for these underlying mechanisms, we present an in-depth analysis of recent clinical trials. We evaluate the evidence from large-scale cardiovascular outcome trials of anti-inflammatory agents like colchicine, alongside mechanism-based trials of vasodilators, novel cell-based therapies, and other targeted agents specifically in the CMVD population. This clinical evidence highlights the therapeutic promise of immunomodulation while underscoring the limitations of empirical, non-stratified treatment approaches. Finally, we posit that future progress depends on precision medicine frameworks that integrate neuro-immune profiling with mechanism-based patient stratification. By synthesizing contemporary mechanistic understanding and robust clinical evidence, this review advances novel diagnostic and therapeutic perspectives through a neuro-immunological lens.

## Graphical Abstract

**Figure:**
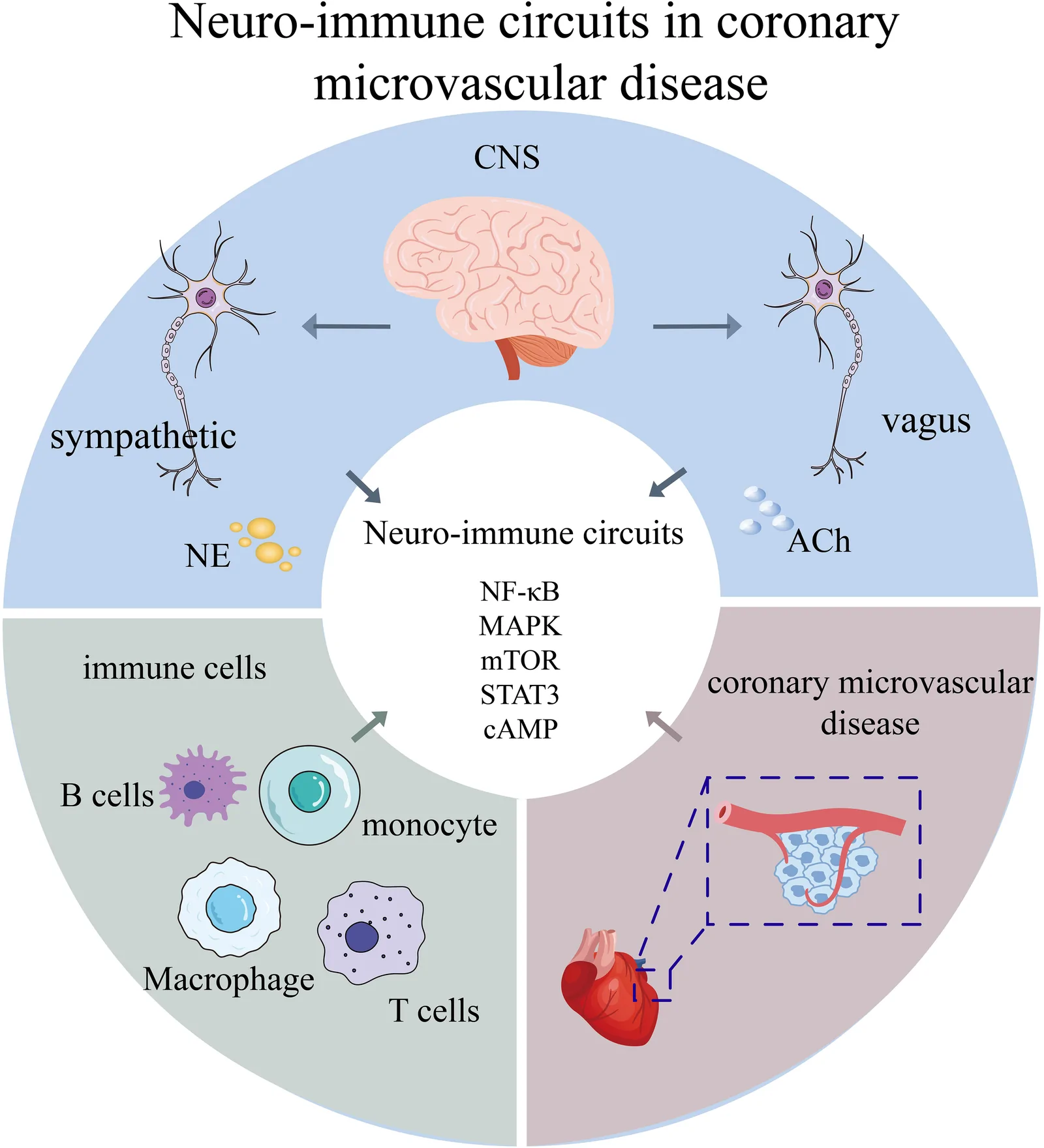

## 1 Introduction

Coronary microvascular disease (CMVD) is a clinical condition characterized by objectively verified myocardial ischemia or exertional angina, resulting from structural and/or functional abnormalities in the coronary microvasculature triggered by various underlying mechanisms (1). Despite its clinical relevance, epidemiological data on CMVD in large population cohorts remains limited. A 2017 meta-analysis encompassing approximately 1.4 million participants across 54 studies revealed that nearly 67% of patients presenting with angina pectoris and clinical signs of myocardial ischemia exhibited no evidence of obstructive coronary artery stenosis (2). Notably, CMVD occurs more frequently in women than in men. CMVD can be classified into three subtypes based on its etiological features (1): CMVD with non-obstructive coronary arteries (2), CMVD coexisting with obstructive coronary artery disease, and (3) other less common forms. Among these, the non-obstructive subtype includes clinical entities such as microvascular angina (MVA) and the coronary slow flow phenomenon. MVA, also known as cardiac syndrome X, is characterized by anginal symptoms and objective evidence of myocardial ischemia, in the absence of obstructive coronary artery disease, as confirmed by coronary angiography or computed tomographic angiography (CTA). Typically, patients present with exertional or resting angina, a positive treadmill exercise test, and normal or near-normal coronary angiography findings (3). Although MVA was previously considered to be irrelevant to cardiogenic causes and showed a favorable clinical prognosis, recent evidence suggests it associating with increased cardiovascular events and poorer long-term outcomes. Furthermore, patients tend to be driven to seek medical attention due to recurrent angina pectoris symptoms and even undergo multiple coronary angiography examines, imposing not only a heavy healthcare burden but also a seriously affected life quality.

Endothelial dysfunction is widely recognized as a central pathological mechanism in the progression of CMVD, thus it has become a crucial therapeutic strategy to improve it. In recent years, a growing amount of researches have underscored the pivotal role of neuroimmune interactions in preserving vascular endothelial function. Studies have demonstrated a close connection between the immune and nervous systems, with various branches of peripheral nervous system, including sensory, sympathetic, vagal, and enteric neurons, transmitting signals that modulate immune cell activity. Additionally, the central nervous system and neuroendocrine pathways—particularly the hypothalamic-pituitary-adrenal (HPA) axis—have been shown to exert significant regulatory effects on immune responses. Anatomically the vascular wall is composed of three distinct layers: the intima, media, and adventitia. The intimal layer consists of endothelial cells, the middle layer typically elastic fibers and other materials, and the outer layer epithelial cells. Although the intima-media layer is not directly innervated, the peripheral nervous system can establish with the tunica-media layer of blood vessels. Increasing evidence shows a regulatory role that sympathetic nervous system played in the occurrence and progress of arterial inflammation. While atherosclerotic plaques themselves are not innervated by nerves, ablation of sympathetic innervation in these areas can alleviate the formation of atherosclerotic plaques, which is a potential evidence of neuromodulation influencing plaque progression and, leads to a potential new therapeutic strategy (4). Immune cells play critical role in CMVD, and modulating immune responses has been proven effective in preventing atherosclerosis. The immune mechanisms in CMVD involve both innate immunity and adaptive immunity (5). Innate immunity is mediated by immuno-inflammatory cells such as monocytes and macrophages, which respond to the excessive accumulation of lipoproteins. In contrast, adaptive immunity is mediated by specific subsets of antigen-responsive T cells that influence inflammation by releasing various cytokines or antibodies, exerting either anti- or pro-inflammatory effects.

There is a close morphological and functional relationship between nerves and immune systems, which plays a critical role to several physiological processes and pathological conditions throughout the body. Mohanta et al. (2022) demonstrated that in apolipoprotein E-deficient (ApoE^-^/^-^) mice, both sensory and sympathetic nerve fibers are abundantly distributed within the adventitia of large arteries, such as the aorta (6). These axonal networks formed extensive neuroimmune cardiovascular interfaces (NICIs) by interacting with immune cells in artery tertiary lymphoid organs (ATLOs) and along the adventitia–media border. They were also significantly increased in regions adjacent atherosclerotic plaques. Among them, the nervous system exerts control over immune function, i.e. “neuro-immune” regulation, through various neural components, such as peripheral sensory nerves and autonomic nerves (sympathetic and parasympathetic), etc. Modulating immune organs and barriers—such as spleen, adrenal gland, skin, respiratory, and gastrointestinal systems—may present a potential novel clinical strategy for enhancing systemic immune function.

The immunological and neurological systems maintain a sophisticated, bidirectional communication network that dynamically regulates physiological homeostasis. This neuroimmune crosstalk is facilitated by specialized neuroimmune cell units–functional anatomical complexes where immune cells and neurons colocalize to execute coordinated biological functions. Beyond local interactions, the central nervous system engages in long-distance communication with peripheral immune cells through neural, endocrine, and paracrine signaling pathways (7). The nervous system modulates immune-inflammatory homeostasis through neural and endocrine pathways by releasing various cytokines, neurotransmitters, and neuropeptides, while the immune system reciprocally modulates neural activity through immune cells that both secrete inflammatory mediators and express neurohormonal receptors (e.g., angiotensin II and adrenergic receptors) capable of binding neural signals. This intricate tripartite interaction among the nervous, immune, and cardiovascular systems constitutes a fundamental pathophysiological axis, with growing evidence establishing the neuroimmune network as a critical determinant in both the pathogenesis and progression of CMVD, particularly through its roles in mediating endothelial dysfunction, microvascular remodeling, and ischemic pathophysiology. Recent studies have proposed a neurobiology-centered framework known as the “neuroimmune cardiovascular circuit hypothesis”. According to this hypothesis, afferent neural signals originating from the arteries or heart are transmitted to various sensory neurons in the brain. The brain’s primary integration centers receive and integrate inputs from the arterio-cerebral and cardio-cerebral circuits, processing them in conjunction with axonal connections and humoral signals from distant brain regions. These integration centers continuously modify the incoming signals and transmit them to efferent neurons, which in turn relay the information back to the cardiovascular system. Notably, the primary integration centers are connected to and receive input from secondary brain centers, which regulate a wide array of brain functions, including immunological memory, stress-related hormone release, pain perception, and emotional responses. The neuroimmune cardiovascular circuit hypothesis offers a novel perspective for understanding the complexity of cardiovascular diseases.

Recent research has increasingly demonstrated the crucial role of crosstalk between the immune and nervous systems in maintaining the health of coronary microvessels. From a clinical perspective, mechanistic studies in the neuro-immune field have offered potential avenues and new approaches for the diagnosis and treatment of CMVD. In this context, the present article provides a comprehensive review of recent advances in the understanding of neuro-immune interactions in CMVD.

## 2 Neurobiology of the cardiovascular system

### 2.1 Central nervous system

The central nervous system (CNS) plays a fundamental role in regulating cardiovascular function. It maintains a close relationship with the heart and blood vessel via a complex network of neurons. Though the sympathetic and parasympathetic branches of the autonomic nervous system, the CNS control a number of physiological parameters, including blood pressure, heart rate, and blood flow distribution. Studies have shown that various areas of the CNS, including the hypothalamus and brainstem, are capable of detecting hemodynamic changes and integrate cardiovascular responses via neural signaling pathways. Moreover, the CNS regulates cardiovascular function by influencing the endocrine system, including the regulation of hormone release, i.e., epinephrine and norepinephrine (8). This crosstalk between the neuroendocrine system governs not only short-term cardiovascular responses but also contributes to long-term adaptive adjustments, for example, the control of cardiovascular adaptation during physical activity, stress, and pathological states.

### 2.2 Peripheral nervous system

The peripheral nervous system (PNS) relays signals between the CNS and cardiovascular system via sympathetic and parasympathetic nerves. Sympathetic innervation is prominent in cardiac tissues, including myocardium, conduction systems, and heart valves, and exhibits a significant relationship with cardiac function changes.

The intima-media layer of the blood vessel lacks direct innervation, but the PNS connects with the tunica adventitia, contributing to coronary microvascular regulation. Sensory nerves detect physiological factors like blood pressure and oxygen saturation and convey this to the CNS for regulatory response.

Sympathetic nervous system activation induces increased heart rate, enhanced myocardial contractility, and vasoconstriction, ultimately leading to elevated blood pressure. Norepinephrine exerts its effects by binding to β-adrenergic receptors in cardiac tissue and α-adrenergic receptors in vascular tissue. Accumulating evidence has demonstrated that sympathetic overactivation is closely associated with the pathogenesis of several cardiovascular diseases, including heart failure and hypertension. Following myocardial injury, the cardiac sympathetic nervous system undergoes abnormal regeneration, a process referred to as sympathetic remodeling, which includes both denervation and hyperregeneration (7).

Pathogenic factors such as inflammation and myocardial ischemia alter the spatial distribution and density of cardiac sympathetic nerve fibers within the myocardium. This process involves both functional and structural remodeling; functionally, stimuli such as increased cardiac filling pressures and myocardial ischemia can trigger a cardiac-specific sympathoexcitatory reflex, leading to a selective increase in local sympathetic activity (9). Anatomical mapping studies have demonstrated that these changes manifest as a heterogeneous nerve pattern, characterized by denervation in the ischemic core and sympathetic hyperinnervation (or “hyper-sprouting”) in the peri-infarct and remote territories (10, 11).

Evidence has increasingly highlighted the role of sympathetic nervous system activation in modulating inflammatory processes within the coronary vasculature. Mohanta et al. (2022) demonstrated that in ApoE^-^/^-^ mice, the adventitia of arteries is directly innervated by sympathetic and nociceptive (injurious receptor) axon endings. These neural elements interact with immune cells to form neuroimmune cardiovascular interfaces (NICIs), which play a regulatory role in atherosclerosis progression (6).

**Figure 1:**
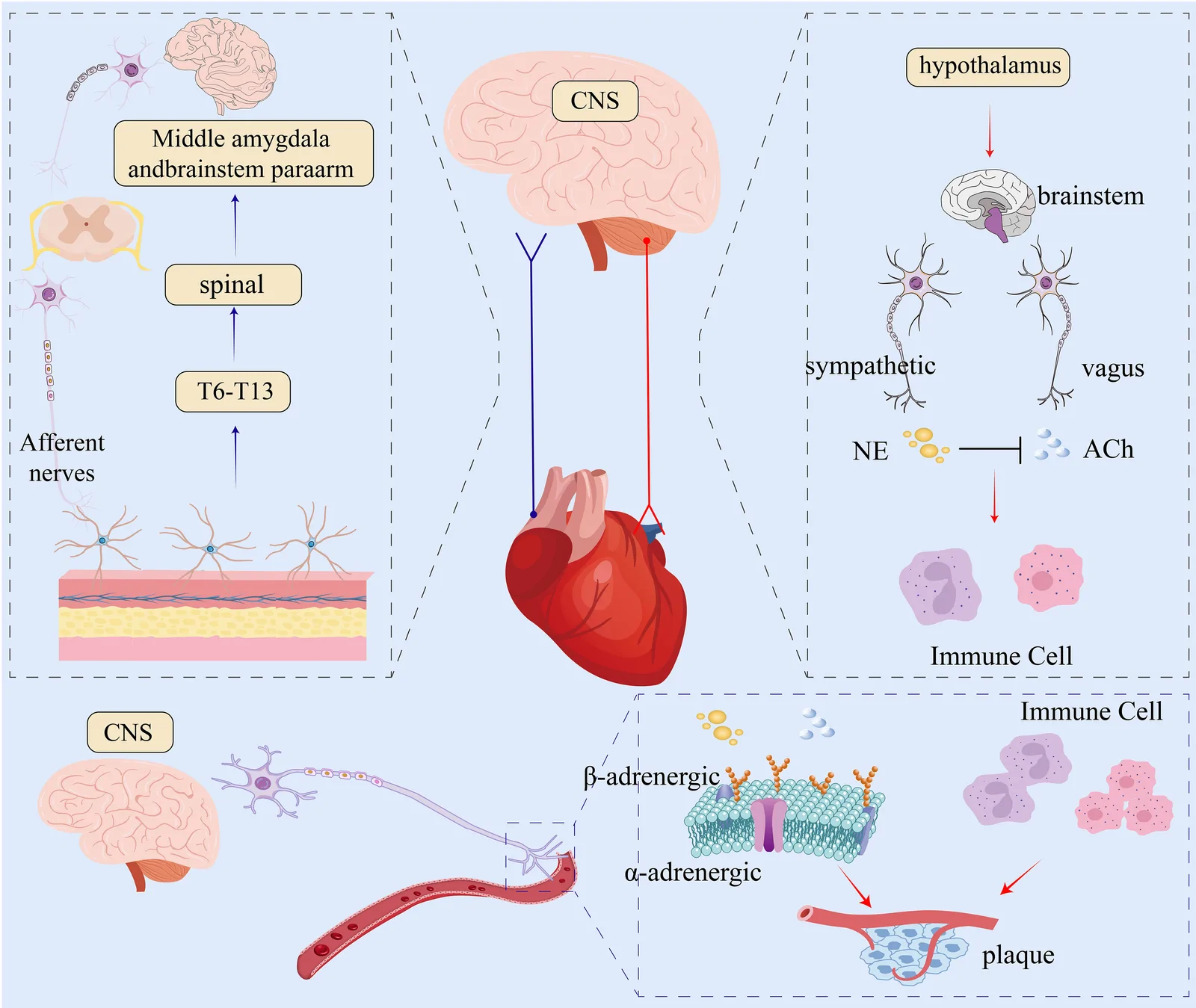
Neurobiological regulation of the cardiovascular system. The central nervous system (CNS) processes afferent signals from the periphery (blue arrow) and sends efferent commands to the heart and vasculature (red arrow) via autonomic pathways. The sympathetic pathway releases norepinephrine (NE) to increase heart rate and vasoconstriction. The parasympathetic (vagal) pathway releases acetylcholine (ACh) to decrease heart rate and can inhibit sympathetic activity, indicated by the black T-bar. Both neurotransmitters modulate immune cells, influencing inflammation and plaque formation. CNS, central nervous system; NE, norepinephrine; ACh, acetylcholine.

Previous studies have shown that increased plaques are positively correlated with extensive neuroinflammation in the PNS (4). Given the dependence of sympathetic nerves on norepinephrine-mediated signaling, it has been tentatively shown that lowering norepinephrine levels or surgically severing the nerve conduction pathways innervating the ependymal membrane decreases the influx of leukocytes into the atherosclerotic plaques due to endothelial cell activation and stress-induced leukocyte influx, and decreases plaque volume (12). In summary, the nervous system can intervene in the inflammatory response and thus participate in the treatment of coronary microvascular disease by regulating immune cells. The neuroanatomical basis and regulatory effects of the sympathetic nervous system on cardiovascular and immune responses are summarized in **Figure 1**, which illustrates norepinephrine-mediated signaling, sympathetic remodeling, and immune-endothelial interaction in coronary microvascular disease.

Operating primarily through the vagus nerve, the parasympathetic nervous system utilizes acetylcholine as its principal neurotransmitter. Stimulation of these nerves normally results in reduction in heart rate, lowered myocardial contractility, and vasodilation, hence reducing blood pressure. Additionally, findings indicate that increased parasympathetic activity may counteract sympathetic excitatory effects and contribute to cardiovascular homeostasis. However, parasympathetic dysfunction is usually accompanied by arrhythmias and increased cardiovascular incidents in heart disease patients.

### 2.3 Cardiovascular-brain circuits

Over the past decade, significant advances have been made in our understanding of the cardiovascular-brain circuit (CBC)—a dynamic and reciprocal communication system integrating the nervous, cardiovascular, and immune systems. Rather than being limited to classical neural conduction, cardiovascular neuromodulation is now recognized to involve a complex neuro-immune-cardiovascular network, comprising two primary subsystems: the arterial-brain circuit (ABC) and the heart-brain circuit (HBC), which exhibit significant cross-talk in maintaining cardiovascular homeostasis (13, 14). These circuits integrate mechanical, inflammatory, and electrophysiological signals from the vasculature and heart, transmitting them to brain regions that coordinate neurohumoral responses (15).

The arterial-brain circuit (ABC) originates from specialized sensory neurons located in the outer arterial wall, which detect atherosclerotic plaque burden and local/systemic inflammation. These nociceptive afferent signals travel via thoracic spinal nerves (T6–T13) to the central nervous system, where they are processed in the spinal cord and subsequently relayed to higher-order brain centers, including the parabrachial nucleus and amygdala in the brainstem (6). The efferent limb consists of sympathetic fibers originating from the medulla oblongata and hypothalamus, descending through the intermediolateral column of the spinal cord, projecting to the coeliac and sympathetic ganglia, and finally innervating the arterial adventitia.

This entire circuit has been visually confirmed in mice using viral tracing techniques, which demonstrated that afferent nociceptive signals from atherosclerotic lesions relay through the T6–T13 spinal segments, project to brainstem regions such as the parabrachial nucleus and amygdala, and that sympathetic efferents return via specific ganglia to modulate the arterial immune environment (6). In this feedback loop, the sensory nervous system functions as the detector, and the sympathetic system acts as the effector, establishing a dynamic neurovascular communication interface.

The ABC exerts direct immune-regulatory functions, influencing both the formation and stability of atherosclerotic plaques. Sympathetic feedback through this circuit can regulate myeloid cell recruitment, cytokine production, and local vascular inflammation. Disruption of this circuitry—through sympathectomy or chemical denervation—has been shown to reduce atherosclerotic burden and promote plaque stability (6). Studies also revealed that neural remodeling, initiated by plaque-induced sensory input, can enhance sympathetic and parasympathetic sprouting in the arterial wall, creating a feedforward loop of inflammation and neurovascular remodeling (6). Experimental interruption of the ABC via ventral ganglionectomy led to a reduction in arterial tertiary lymphoid organ (AL0) number, size, and cellular composition, correlating with smaller, more stable plaques and attenuated vascular inflammation.

The HBC operates primarily through the vagus nerve and sympathetic nervous system, facilitating bidirectional communication between the heart and brain. Mechanical and electrophysiological signals from the heart, such as heart rate and pressure changes, are detected by baroreceptors and cardiac sensory neurons, which transmit information via vagal and sympathetic afferents to brain regions including the hypothalamus and brainstem nuclei (16, 17). The hypothalamus, serving as a central integrator, processes these inputs and coordinates downstream signals through vagal efferents and sympathetic fibers to peripheral tissues (13).

The HBC plays a pivotal role in modulating systemic and localized immune responses. Both sympathetic and vagal pathways establish neuroimmune circuits that regulate inflammatory processes via afferent and efferent signaling. Activation of vagal efferents leads to anti-inflammatory effects, primarily through α7 nicotinic acetylcholine receptors, while sympathetic overactivity promotes myeloid cell recruitment to vascular lesions and enhances systemic inflammation (18).

In patients with CMVD, abnormalities in cardiovascular regulation are closely linked to cognitive impairment and emotional dysregulation. Microvascular damage compromises cardiac perfusion, leading to clinical symptoms such as angina pectoris and heart failure, but also impacts brain function through the heart-brain axis. Within this framework, cardiac-origin signals transmitted via vagal and sympathetic nerves influence brain regions governing mood, behavior, and cognition (14).

## 3 The important role of immune cells in cardiovascular disease

Immune cells play a pivotal role in the development and progression of CMVD by mediating processes such as subendothelial lipid retention, cellular migration, and proteolytic tissue damage (19). These mechanisms lead to chronic arterial wall inflammation and ultimately plaque formation. Supporting this, Coller (2005) showed a strong correlation between a higher leukocyte count and a higher chance of cardiovascular problems. This risk is exacerbated by the process of leukocyte recruitment to tissues and the interaction of adherent leukocytes with other blood components, which can lead to vascular occlusion (20).

Although earlier research implicated macrophages as the primary immune cells involved in cardiovascular disease, subsequent studies have revealed other participating cells, such as mast cells, myeloid cells, and CD4+ lymphoid and regulatory T lymphocytes (21). Immune cells involved in cardiovascular disease are broadly categorized as either innate immune cells (e.g., monocytes, macrophages, neutrophils, among others) or adaptive immune cells (e.g., helper T1, Th2, Th17, CD8+, NK, and regulatory T cells) (22). The equilibrium between pro- and anti-inflammatory reactions is also regulated by these adaptive cells. Key signaling pathways governing immune cell function include the Toll-like receptor (TLR), mitogen-activated protein kinase (MAPK), nuclear factor-κB (NF-κB), reactive oxygen species (ROS), CD40–CD40L, inflammasome–caspase, and peroxisome proliferator-activated receptor (PPAR) pathways (23).

### 3.1 Intrinsic immune cells

Neutrophils are the first effector cells activated during the acute inflammatory response, serving as primary responders to tissue injury or infection and exacerbating inflammation through pro-inflammatory mediator release. Under conditions of sustained inflammatory conditions, their continuous accumulation and secretion of pro-inflammatory granule proteins like MPO, defensins, and CRISP3 contribute to tissue damage and chronic inflammation (24). Specific mechanisms include MPO’s role in facilitating LDL nitration and lipid peroxidation, promoting foam cell formation via macrophage ox-LDL uptake, and neutrophil-derived protease-associated lipid transfer proteins enhancing MMP-9’s matrix-degrading capacity by preventing its inactivation. Neutrophils also interact with endothelial cells, secreting tissue factor and chemokines that recruit more neutrophils and intensify inflammation (25). Evidence points to a significant association between neutrophils and the development and instability of atherosclerotic plaques (26). Clinical research shows that in individuals with coronary artery disease, neutrophil counts are directly connected with the severity of coronary lesions (27).

During persistent inflammation, monocytes differentiate into transitional effector cells that play a critical role in sustaining the immune response. Primarily derived from the bone marrow, monocytes circulate between the bloodstream and peripheral tissues. Their migration to vascular lesion sites is guided by chemokine signals like CCR2–CCL2 and CCR5–CCL5. Studies using animal models have demonstrated that elimination of these chemotactic signals can slow down atherosclerosis (28). Acute inflammation enables monocytes to mature into macrophages early, which enhances local phagocytosis and immunologic defense. Nevertheless, during immune over-activation, monocyte function can be impaired, advancing cardiovascular disease development (29).

Macrophages play a central role in chronic inflammation, ensuring tissue repair and immune homeostasis. In atherosclerotic lesions, macrophages originate from two main sources: resident vascular macrophages and monocytes that differentiate from circulation (30). Macrophage activation and polarization phenotypes, with M1 and M2 subtypes being more prevalent, are determined by the local microenvironment of the plaque and cytokine signals. Exposure to lipopolysaccharide (LPS) and interferon-gamma (IFN-γ) activates M1 macrophages, sometimes referred to as classically activated macrophages, resulting in the release of pro-inflammatory cytokines such interleukin-6(IL-6), interleukin-12(IL-12), and tumor necrosis factor-alpha (TNF-α). The cells contain a wide variety of pattern recognition receptors (PRRs) and begin inflammatory cascades on recognizing oxidized low-density lipoprotein (ox-LDL) and related substances (31, 32). In contrast, M2 macrophages, often referred to as alternatively activated macrophages, chiefly release anti-inflammatory cytokines (such as IL-10, TGF-β) and take part in immunoregulation and tissue remodeling (33).

Natural killer (NK) cells, a subset of lymphocytes, express receptors that are highly sensitive to cellular stress and pathological alteration. They exert potent cytotoxic effects by releasing perforin, granzymes, and/or cytokines such as IFN-γ. Chemokines such as MCP-1, and fractalkine, and cytokines, including IL-15, and IFN-γ, induce NK cell activation, cytotoxicity, and migration to atherosclerotic plaques. Upon activation, NK cells produce addition IFN-γ, which contributes to immune responses that promote atherosclerosis (34).

### 3.2 Adaptive immune cells

Adaptive immunity offers antigen-specific defense mechanisms and long-term immunological memory. In atherosclerosis, early activation of innate cells like monocytes and macrophages subsequently triggers adaptive immune responses, where T and B cells play pivotal roles.

One of the primary immune cell types drawn into atherosclerotic plaques are activated T cells, which also play a significant role in the development of the disease. Studies have shown that ApoE^-/- mice lacking both T and B cells develop less severe atherosclerotic lesions, whereas adoptive transfer of LDL-specific CD4^+ T cells into immunodeficient ApoE^-/- mice aggravates lesion development (35). Key T cell subsets in atherosclerosis, such as Th1, Th17, and T follicular helper (Tfh) cells, secrete distinct cytokines and exert divergent biological functions, forming a complex regulatory network governing plaque formation and progression.

Th1 cells are activated by antigens like ox-LDL and native LDL, releasing cytokines that promote inflammation (e.g., IFN-γ, TNF-α) that promote atherogenesis and vascular inflammation. IFN-γ is a major pro-atherogenic cytokine that exacerbates endothelial dysfunction, enhances monocyte recruitment, promotes macrophage activation, and facilitates foam cell formation (36).

Th17 cells and their signature cytokine IL-17A have been increasingly implicated in the pathogenesis of atherosclerosis, yet their precise role remains controversial. On the one hand, compelling evidence suggests that IL-17A promotes atherogenesis by inducing vascular endothelial cells to produce various chemokines, including CCL2, CXCL1, and CXCL5, which enhance leukocyte recruitment and amplify local inflammation. For instance, Lin et al. (2022) demonstrated that IL-17A-expressing Th17 subsets in the aorta induced endothelial chemokine production, directly linking Th17 activity to inflammatory cell infiltration and lesion development (37). Similarly, Robert et al. (2022) reviewed how IL-17A stimulates endothelial and smooth muscle cells to express adhesion molecules and pro-inflammatory mediators, further contributing to vascular dysfunction (38). However, this pro-atherogenic narrative is not universally supported. Some studies report that IL-17A may also exert protective effects, such as promoting fibrous cap formation and plaque stability under specific conditions (39). Moreover, Xu et al. (2020) emphasized the plasticity of Th17 cells, whose inflammatory or regulatory functions are shaped by their cytokine milieu, particularly by anti-inflammatory mediators like TGF-β and IL-10 (40). Supporting this nuance, Taleb et al. (2015) highlights in their comprehensive review that IL-17’s function in atherosclerosis is highly context-dependent, varying by genetic background, disease stage, and experimental conditions (41). While some murine models show IL-17A promotes lesion development, others suggest neutral or even protective roles, reflecting a complex interplay of immune regulation and environmental cues. This discrepancy likely stems from several factors. First, the effects of Th17/IL-17 signaling are modulated by the surrounding immune environment; for example, IL-10 can skew Th17 responses toward a less pathogenic phenotype. Second, murine models differ significantly in genetic and inflammatory profiles, which may not accurately reflect human pathology. Third, IL-17 may exert temporally distinct effects: pro-inflammatory in early lesion development and potentially reparative or stabilizing in later stages.

Regulatory T cells (Tregs) are well-established immunomodulators with anti-atherosclerotic properties. Tregs secrete IL-10 to suppress inflammatory processes and maintain immune tolerance. Reduced Treg activity or IL-10 production has been associated with increased local inflammation and enhanced atherosclerotic burden (42).

**Figure 2:**
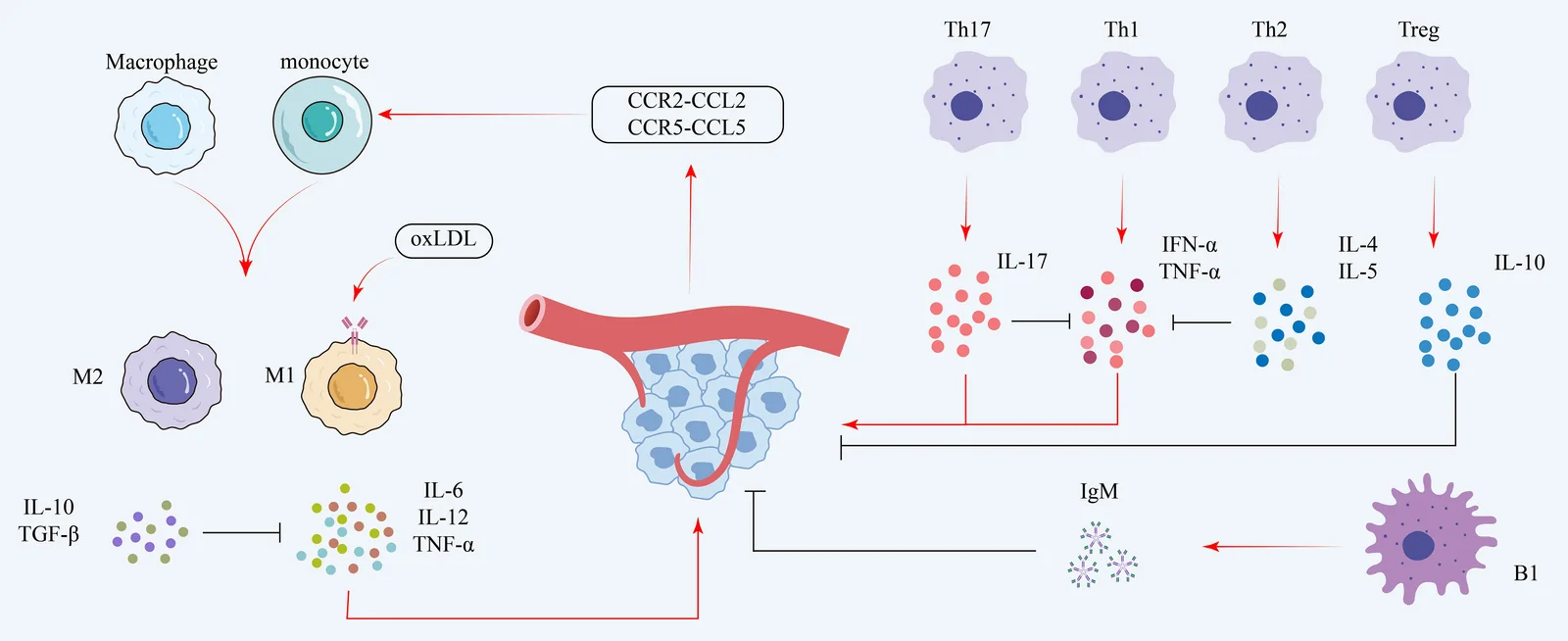
Immune mechanisms in coronary microvascular disease. The diagram illustrates stimulatory pathways (red arrows) and inhibitory pathways (black T-bars). Monocytes are recruited via chemokines (e.g., CCR2-CCL2) and polarized by oxidized LDL (oxLDL) into pro-inflammatory M1 macrophages (secreting IL-6, TNF-α). T helper (Th)1 and Th17 cells further promote inflammation. These pro-inflammatory effects are countered by M2 macrophages, Th2 cells, regulatory T (Treg) cells, and B1 cells, which release protective, anti-inflammatory mediators such as IL-10, TGF-β, and IgM. oxLDL, oxidized low-density lipoprotein; IL, interleukin; TNF-α, tumor necrosis factor-alpha; TGF-β, transforming growth factor-beta; Th, T helper; Treg, regulatory T cell; IgM, immunoglobulin M; CCR, C-C chemokine receptor; CCL, C-C motif chemokine ligand.

B cells contribute to both innate and adaptive immunity by secreting cytokines and antibodies, in addition to their roles in adaptive responses. They promote the progression of atherosclerosis through antibody-mediated responses targeting ox-LDL and by activating T cells (43). In contrast, B1 cells, a subset of B lymphocytes, secrete natural IgM antibodies. By preventing the production of foam cells, this activity prevents atherosclerosis (44). **Figure 2** summarizes the contributions of macrophage polarization and T/B lymphocyte-derived cytokines to endothelial inflammation and immune cell recruitment in coronary microvascular disease.

## 4 Interaction between the nervous system and immune cells

### 4.1 Systemic and regional immunomodulation by sympathetic nerves

On the one hand, sympathetic nerves are directly involved in the stress response, through innervation of adrenal medullary chromaffin cells, releasing catecholamine transmitters such as epinephrine, norepinephrine, and dopamine into the blood circulation, thus realizing the regulation of organismic and systemic immune functions; on the other hand, sympathetic nerves innervate almost all organs and tissues, releasing transmitters such as norepinephrine and neuropeptide Y through the endings to realize the target organs and regional immune regulation (45–47). Sympathetic release of norepinephrine able to attach toα- or β-adrenergic receptors on a variety of immune cells, affecting their migration, differentiation, and cytokine synthesis (48–50). Due to differences in transmitter concentrations, receptor expression levels, and inflammatory response processes, sympathetic regulation of immune function exhibits bidirectional regulation (51). For example, lower concentrations of norepinephrine (NE) have a stronger affinity for a-receptors and reduce intracellular Cyclic Adenosine Monophosphate (cAMP) levels, whereas higher concentrations of NE bind more strongly to β-receptors and cause an increase in intracellular cAMP levels (52); activation of B receptors on CD4+ T cells promotes their differentiation and synthesis of cytokines (53). However, macrophage’s production and release of TNF-α are inhibited when their B receptors are activated (54); Moreover, localized inflammatory lesions in the target organ can, to a certain extent, directly affect the normal morphology and functional activities of the sympathetic nerves, and thus the interaction between sympathetic nerves and the target-organ immunity is a dynamically regulated process (55).

### 4.2 Vagus nerve-mediated “inflammatory reflex” exerts extensive immunomodulation

In addition to its role in regulating visceral smooth muscle contraction, glandular secretion, and blood flow, the vagus nerve mediates an immunomodulatory mechanism known as the “inflammatory reflex”, primarily operating through the vagovagal circuit (56). Its anti-inflammatory capacity is primarily executed via the cholinergic anti-inflammatory pathway (CAIP) and various non-canonical regulatory pathways.

In the canonical pathway, pathological stimuli such as bacterial endotoxins trigger peripheral immune cells—primarily macrophages—to release large quantities of pro-inflammatory cytokines. Afferent vagal fibers detect these signals and transmit them to the central nervous system, which in turn stimulates efferent vagal pathways to secrete acetylcholine (ACh). Acetylcholine interacts with α7 nicotinic acetylcholine receptors (α7nAChR) present on immune cells, leading to the downregulation of pro-inflammatory cytokines, including TNF-α and IL-6 (57, 58). According to findings by Yang et al. (2022), α7nAChR is essential for the anti-inflammatory action exerted by the vagus nerve in response to lipopolysaccharide-induced cytokine storms. The diminished anti-inflammatory response to famotidine in mice lacking α7nAChR highlights the importance of this receptor and the vagal pathway in conventional inflammatory modulation (59).

Beyond the classical CAIP, the vagus nerve also modulates inflammation via non-canonical pathways, which do not rely on the spleen or α7nAChR-mediated signaling. According to de Araujo and de Lartigue (2020), maintaining intestinal immune stability is significantly governed by the hepatic branch of the vagus nerve (60). Experimental data indicate that selective hepatic vagotomy exacerbates colitis in mice (61), suggesting that this regulatory circuit may influence immune responses through the modulation of regulatory T cell (Treg) differentiation.

Recent investigations have brought attention to the involvement of intestinal microbiota in modulating the vagus nerve–mediated inflammatory reflex. Commensal microbes can communicate with the vagus nerve through microbial metabolites or mimic signaling, thereby impacting the central–peripheral neuroimmune axis and systemic inflammation (60). In addition, agents such as famotidine—an inhibitor of H2 histamine receptors—have been found to engage the vagus nerve–associated inflammatory circuit and suppress cytokine storms elicited by lipopolysaccharide (59), supporting the prospective treatment value of vagus-targeted interventions in inflammatory disorders.

Among the cytokines regulated by the vagus nerve, TNF-α serves as a critical factor in orchestrating the inflammatory cascade. Its expression is significantly influenced by vagal signaling via the CAIP. Specifically, vagal stimulation promotes ACh release, which binds to α7nAChR on immune cells and inhibits NF-κB, a principal transcription factor essential for driving TNF-α and other cytokine gene transcription (62, 63). In animal models with vagotomy, TNF-α levels were markedly elevated, underscoring the importance of intact vagal integrity in inflammatory control. In contrast, stimulation of the vagus nerve (VNS) in LPS-challenged murine models significantly reduced TNF-α concentrations, further substantiating the anti-inflammatory role of this neural pathway (58).

High mobility group box 1 (HMGB1), a non-canonical mediator secreted during infection or tissue trauma, is also subject to vagal regulation in the resolution stages of inflammation. Vagal stimulation significantly reduces HMGB1 release, and this could be mediated, at least in part, via α7nAChR signaling. Furthermore, the vagus nerve has been implicated in the downregulation of other pro-inflammatory cytokines (63, 64), indicating its broad regulatory role within the inflammatory network. Taken together, these findings underscore the pleiotropic immunomodulatory effects of vagal signaling in systemic inflammation.

As a promising modality in the field of bioelectronic medicine, VNS presents a novel therapeutic avenue. Nevertheless, the underlying mechanisms, as well as its long-term safety and efficacy, warrant further exploration in future studies.

### 4.3 Immunomodulation of peripheral sensory nerves through peptide transmitters

C-fiber sensory neurons, also known as peptidergic neurons, situated in the trigeminal or dorsal root ganglia, are not only capable of receiving external injurious mechanical, temperature, and chemical stimuli, but are also activated by relevant factors involved in infectious and inflammatory responses (65–67). These sensory nerve fiber endings exhibit widespread presence in the mucous membranes of the skin, lungs, and gastrointestinal tract and exist in close proximity to local lymphoid tissues and immune cells. When the mucosal barrier is damaged, class C nerve fiber endings release peptide transmitters such as SP (Substance P) and CGRP (Calcitonin Gene Related Peptide), which act on the corresponding receptors on the adjacent immune cells to regulate the local immune microenvironment (66, 68–72). In addition to their direct modulation of local immune responses, these sensory nerve fibers also demonstrated the capacity to dampen systemic inflammatory responses during situations of endotoxemia (73–76). The observation suggests that the neural pathways involved with immune regulation can recruit a number of circuits, among them peripheral reflex mechanisms and central processing.

### 4.4 Immune cell interactions and regulation in the nervous system

Microglia and T cells are the major populations of immune cells present within the CNS, whose overall roles are to maintain neural homeostasis and modulate neurological function. In addition to their immune surveillance and host defense role, microglia are involved in neuroprotective, synaptic reorganization, and tissue repair functions. Upon pathogen detection or tissue damage, microglia are rapidly activated by pattern recognition receptors and release a vast variety of cytokines to coordinate local immune response. For instance, pro-inflammatory mediators interleukin-1 beta (IL-1β) and TNF-α induce the recruitment of accessory immune cells, eosinophils, and neutrophils to infection- or injury-susceptible areas for antimicrobial defense (77).

Apart from their immunological functions, microglia also release neurotrophic factors including brain-derived neurotrophic factor (BDNF) and IL-4 that are jointly indispensable for neuronal survival and regeneration. IL-4, for instance, has been reported to increase neuronal growth and survival, and is of extreme importance to cognitive activities like learning and memory (77, 78). IL-4 and IFN-γ are both significant regulators of neuronal transmission. IL-4 deficiency causes extreme impairments of mouse learning behavior, whereas exogenously given IL-4 has the potential to reverse these impairments, demonstrating the critical role played by cytokines in cognitive function (77). Microglial modulation is also involved in following neuroregeneration injury by the release of neurotrophic factors while at the same time suppressing excessive inflammatory activities (79). In neurodegenerative disorders like Alzheimer’s disease, microglial activation might become polarized to a pro-inflammatory phenotype to further increase neuronal injury in a pattern closely correlated with β-amyloid accumulation (78).

Aside from their contribution to microglial activation, T cells—and crucial players within the adaptive immune system—are themselves important determinants of central nervous system (CNS) physiology. Activated CD4^+^ T cells secrete a variety of cytokines that impact both immune as well as neural function. For example, IL-2 secreted by CD4^+^ T cells, aside from stimulating other immune cells to proliferate, increases microglial activation, thus playing a pivotal role within localized immunity (77). IFN-γ, a cytokine secreted by CD4^+^ T cells, increases microglial antimicrobial effects, hence allowing for the elimination of microorganisms as well as potentially for triggering localized inflammatory responses (80, 81). Interestingly, IL-4, a cytokine secreted by Th2-polarized CD4^+^ T cells, has been shown to promote neuronal survival as well as regeneration, reducing cerebral injury caused by ischemia as well as trauma (82, 83). Conversely, IL-10, a secreted anti-inflammatory cytokine by some T cells, is crucial for its neuroprotective action. IL-10 reduces neuroinflammation by suppressing microglial release of pro-inflammatory mediators including TNF-α as well as IL-1β, thus ensuring resolution of inflammation after injury as well as neuronal survival (84, 85).

**Figure 3:**
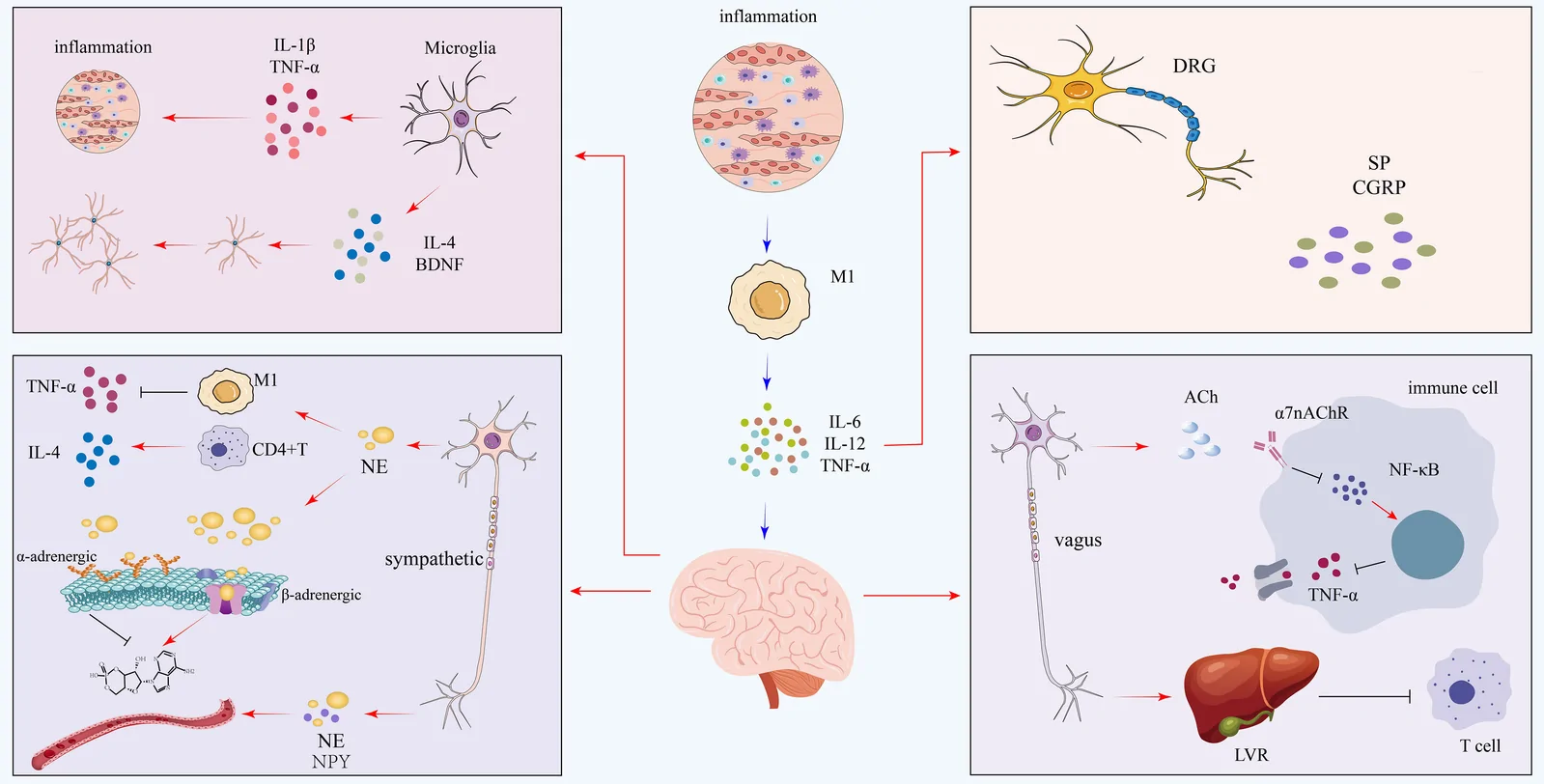
Integrated neuroimmune circuits in coronary microvascular disease. This diagram illustrates how a central inflammatory state, driven by M1 macrophages, is modulated by diverse neural circuits. Pro-inflammatory signals are amplified by sensory neurons of the dorsal root ganglion (DRG), which release substance P (SP) and CGRP, and by the sympathetic nervous system, which uses norepinephrine (NE) and neuropeptide Y (NPY) to promote M1 activation. This is counterbalanced by robust anti-inflammatory mechanisms: the parasympathetic vagus nerve releases acetylcholine (ACh) to suppress TNF-α via the α7nAChR pathway. Notably, microglia in the central nervous system exhibit a dual role, capable of releasing both pro-inflammatory (IL-1β, TNF-α) and anti-inflammatory (IL-4, BDNF) mediators. IL, interleukin; TNF-α, tumor necrosis factor-alpha; DRG, dorsal root ganglion; SP, substance P; CGRP, calcitonin gene-related peptide; NE, norepinephrine; NPY, neuropeptide Y; ACh, acetylcholine; α7nAChR, alpha-7 nicotinic acetylcholine receptor; BDNF, brain-derived neurotrophic factor.

CD8^+^ T lymphocytes perform a vital function for the elimination of tumor cells and infected cells through the discharge of cytotoxins such as TNF-α and perforin. However, their activation under certain situations may unintentionally destroy normal neural tissues (77). In addition, cytokine-driven communication between neurons and immune cells plays an important role in cognitive function as well as synaptic plasticity. For example, IL-4 facilitates synaptogenesis and learning ability (86), whereas IL-6, which is mainly pro-inflammatory, can compromise neuronal function and plasticity by promoting chronic inflammatory signaling (87). Such context-dependent functions of cytokines are inherent to T cell activation and function in diverse physiological and pathological states. These interactions converge into a multi-circuit neuroimmune regulatory framework that influences immune polarization, cytokine expression, and inflammation. **Figure 3** presents a comprehensive model summarizing these coordinated pathways.

## 5 Neuro-immunity and coronary microvascular disease

### 5.1 Association of neuro-immunity with cardiovascular disease risk factors

Atherosclerotic plaques, an important risk factor for cardiovascular disease, consist of a progressive increase in cholesterol, fibrous tissue, and immune cells. When atherosclerotic plaques form, aggregates of immune cells also form in the outer connective tissue. These aggregates have similarities to lymph nodes and contribute to the modulation of immune activity. At the same time the connective tissue around the coronary arteries is rich in nerve fibers, and this outer tissue is used by the nervous system as a major conduit to all organs throughout the body. Upon reaching the brain, signals originating from the plaque influence the autonomic nervous system through vagal pathways, ultimately extending to the spleen. Specific immune cells are activated and enter the circulation, leading to plaque progression. Clinical epidemiological research has indicated that mental stress is a significant contributor to the risk of atherosclerosis and that increased mental stress can drive vascular inflammation and atherosclerotic plaque progression by activating cell adhesion factors on endothelial cells to enhance leukocyte recruitment to tissues (88). Meanwhile, long-term mental stress can also lead to elevated serum cholesterol levels and induce hyperlipidemia. Accordingly, mitigating psychological stress and enhancing mental well-being represent emerging therapeutic strategies in the prevention of stress-related cardiovascular disease.

In addition to mental stress, there is growing evidence that the relationship between injurious feelings and the immune system can further contribute to accelerating the inflammatory process through the mechanisms described above. Improvement of the mental state of individuals with cardiovascular diseases is an evermore integral element of care and is, in turn, closely linked with favorable clinical endpoints. Modulation of neurotransmitters, such as norepinephrine, as well as targeting multifunctional molecules such as Netrin-1, have been proven to be an innovative strategy in the therapy of cardiovascular diseases. In the framework of the development of atherosclerosis, neuroimmune cardiovascular interfaces (NICI)—created from interactions among nerve fibers, immune cells, and vascular endothelium—are shown to play a part in the generation of active plaque structures, such as the ABC complex, in the course of the atherogenic process. It is remarkable that peripheral nervous system axons in areas of atherosclerosis release epinephrine and other neurochemical signals into the vascular epithelium, which may trigger local recruitment and accumulation of immune cells, hence modulating lesion growth. Thus, a local model of stimulation of immune cells by abnormal electrical activity projected from the CNS and activation of β2-adrenoceptors may underlie the neuroimmune axis that regulates atherosclerosis. The targeted regulatory role of genetic techniques also provides some direction for neuroimmunologic approaches to prevent and delay atherosclerotic plaque formation and progression.

An increasing body of experimental evidence highlights the central role played by the neuroimmune system in the etiology of cardiovascular disorders. Of particular interest, it has been shown to play roles in the causation and perpetuation of pulmonary arterial hypertension (PAH), mainly through coordinated modulation of immune function and autonomic nervous system activity. Transcriptomic analyses of PAH models reveal upregulation of key inflammatory mediators, including TNF-α and TGF-β, in addition to the NF-κB signaling pathway being activated. These molecular changes contribute to pulmonary vascular remodeling and disease progression. Concurrently, excessive activation of the sympathetic nervous system (SNS) has been shown to exacerbate disease severity, whereas pharmacological or genetic suppression of SNS activity can alleviate pulmonary artery pressure and improve cardiac function (89).

Additionally, recent studies have identified a dynamic interplay between the heart and neuroimmune circuits in the context of hypertension, particularly through a functional “heart–brain–spleen axis” that modulates cardiac remodeling. In the framework of hypertension, pressure overloading of the heart stimulates the activation of the sympathetic nervous system, which, in turn, activates dorsal vagal complex and splenic innervation, driving placental growth factor secretion (PlGF). PlGF subsequently stimulates the proliferation of NRP1-expressing cardiac macrophages, thereby facilitating adaptive cardiac remodeling. Disruption of this neuroimmune axis, either through neural inhibition or blockade of the PlGF–NRP1 signaling cascade, impairs the heart’s compensatory remodeling capacity and increases susceptibility to heart failure (90). Clinically, circulating PlGF levels have been positively correlated with the degree of myocardial hypertrophy, further supporting the functional relevance of neuroimmune mechanisms in hypertension-induced cardiac pathology.

### 5.2 Molecular mechanisms and diagnostic potential of neuro-immunity in the development of coronary microvascular disease

Recent advances have led to a surge in research have started to concentrate on the role of neuro-immune interactions in coronary microvascular disease, especially the regulatory mechanisms in microvascular endothelial dysfunction, vascular remodeling and microvascular inflammatory responses. For example, increased sympathetic nerve activity and immune responses may activate vascular endothelial cells, smooth muscle cells, and inflammatory responses, exacerbating microvascular damage and affecting microvascular function. Exploring the molecular mechanisms of neuro-immune interactions will enable a deeper comprehension of the pathogenesis of coronary microvascular disease and provide potential targets for clinical diagnostic and therapeutic programs and novel drug development.

#### 5.2.1 Key signaling pathways in the neuro-immune network

The nuclear factor kappa-B (NF-κB) pathway is a critical transcriptional regulator in the pathophysiology of CMVD, orchestrating inflammatory responses in both immune and vascular cells. In cardiac ischemia, inflammatory cytokines such as Interleukin-34 can activate both the canonical (p65) and non-canonical (p52/RelB) NF-κB pathways within macrophages (91). This activation drives the transcription of chemokines like CCL2, which facilitates further macrophage recruitment and polarization, thereby exacerbating cardiac remodeling (91). Separately, the non-canonical pathway is also aberrantly activated directly within the microvasculature itself. In human atherosclerotic lesions, high levels of the initiating kinase, NIK, are found specifically in microvascular endothelial cells (92). This endothelial activation of the non-canonical pathway is associated with increased local inflammation, an unstable plaque phenotype, and the expression of another leukocyte-recruiting chemokine, CXCL12 (92). Therefore, it is plausible that in CMVD, NF-κB acts as a central hub for a self-perpetuating inflammatory cycle, where its activation in both resident immune cells and the endothelium drives chemokine production, leading to persistent immune cell infiltration and microvascular damage.

The Mitogen-Activated Protein Kinase (MAPK) cascade is a key signaling pathway in neuro-immune regulation and is strongly implicated in cardiovascular pathophysiology (93, 94). In the context of cardiovascular disease, such as heart failure, compelling evidence from animal models demonstrates that MAPK activation within central autonomic regulatory areas, including the subfornical organ (SFO) and paraventricular nucleus (PVN), correlates with increased central inflammation, elevated endoplasmic reticulum stress, and enhanced sympathetic outflow (95). Concurrently, in peripheral cardiovascular tissues, pathological stimuli relevant to microvascular disease, such as hyperglycemia, are known to activate MAPK pathways and drive oxidative stress (96). This activation occurs in various cell types including endothelial cells, vascular smooth muscle cells (SMCs), and macrophages, contributing to local inflammation (Wang et al., 2025). Specifically, MAPK signaling in SMCs drives the proliferation that leads to adverse vascular remodeling, while in macrophages its activation is essential for the foam cell formation seen in atherosclerosis (93). These combined peripheral processes ultimately promote endothelial dysfunction (94, 96). Therefore, it is plausible that in CMVD, the MAPK pathway contributes to the disease through a dual mechanism: centrally by promoting autonomic dysregulation, and peripherally by mediating local microvascular inflammation and damage. The observation that central blockade of the p44/42 MAPK branch can blunt sympathetic outflow and decrease systemic inflammation points to its potential as a therapeutic target (95).

While the role of immunity in CMVD is well-established, the specific role of the mechanistic target of rapamycin (mTOR) pathway requires further investigation—for its function as a central coordinator of cellular metabolism and proliferation (97, 98). In the context of cardiovascular disease, mTOR signaling exhibits a complex duality—contributing to both physiological tissue repair and pathological processes. On one hand, chronic or aberrant activation of mTOR, particularly the mTORC1 complex, is implicated in vascular pathology. In states of overnutrition and Type 2 Diabetes Mellitus (T2DM)—both significant risk factors for CMVD—sustained mTORC1 activity can induce a negative feedback loop that suppresses insulin signaling, contributing to metabolic dysfunction (99). This over-activation also drives a maladaptive immune response by promoting pro-inflammatory M1 macrophage polarization and modulating T-cell differentiation, thereby fostering a state of vascular inflammation (97). On the other hand, mTOR signaling is also essential for repair processes. For example, in the nervous system, mTOR activation is crucial for promoting axonal regeneration after injury, though its hyper-activation in astrocytes can paradoxically inhibit repair by causing glial scarring (98). Critically, a study on coronary collateral growth demonstrated that inappropriate *inhibition* of mTOR, downstream of mitochondrial oxidative stress and AMPK activation, can also be detrimental by impairing necessary endothelial tube formation and vascular growth (100).These multifaceted findings suggest that mTOR’s role in the neuro-immune-vascular axis of CMVD may be that of a critical rheostat. It is plausible that in CMVD, a state of metabolic stress leads to chronic, pathological mTORC1 over-activation in immune and vascular cells, while concurrent mitochondrial dysfunction impairs necessary mTOR-dependent repair and angiogenic processes in endothelial cells. Given mTOR’s potent regulatory capacity within the nervous system, it could plausibly coordinate a pathological crosstalk between perivascular nerves and the local vascular immune environment in CMVD.

The signal transducer and activator of transcription 3 (STAT3) pathway is a central node in regulating both immune cell behavior and endothelial signaling. Its role in cardiovascular disease is multifaceted, demonstrating a critical duality between pathological and reparative functions. On one hand, sustained hyper-activation of STAT3 drives adverse cardiovascular remodeling. For instance, stimuli like Angiotensin II can induce a biphasic activation of STAT3 in cardiomyocytes; an initial phase triggers the production of the pro-inflammatory cytokine IL-6, which then establishes a sustained second wave of STAT3 activation that promotes the expression of fibrotic and hypertrophic genes (101). Fundamentally, within the immune system, STAT3 activation is known to orchestrate the differentiation and function of multiple immune cells, and its dysregulation can foster a pro-inflammatory or immunosuppressive microenvironment (102). However, STAT3 signaling is not exclusively detrimental; it is also essential for vascular repair. A study using endothelial progenitor cells from patients with CAD demonstrated that proper activation of STAT3 is essential for VEGFA-mediated angiogenesis in response to ischemic stimuli. Impaired STAT3 function in these cells was directly associated with a reduced capacity for neovascularization (103). This suggests that the balance of STAT3 signaling is crucial. Therefore, it is plausible that in CMVD, the chronic inflammatory and ischemic microenvironment leads to a dysregulation of STAT3 signaling. A sustained, pathological activation could drive microvascular inflammation and remodeling, while an impairment of its reparative, angiogenic functions could compromise the microvasculature’s ability to heal, leading to progressive damage.

**Figure 4:**
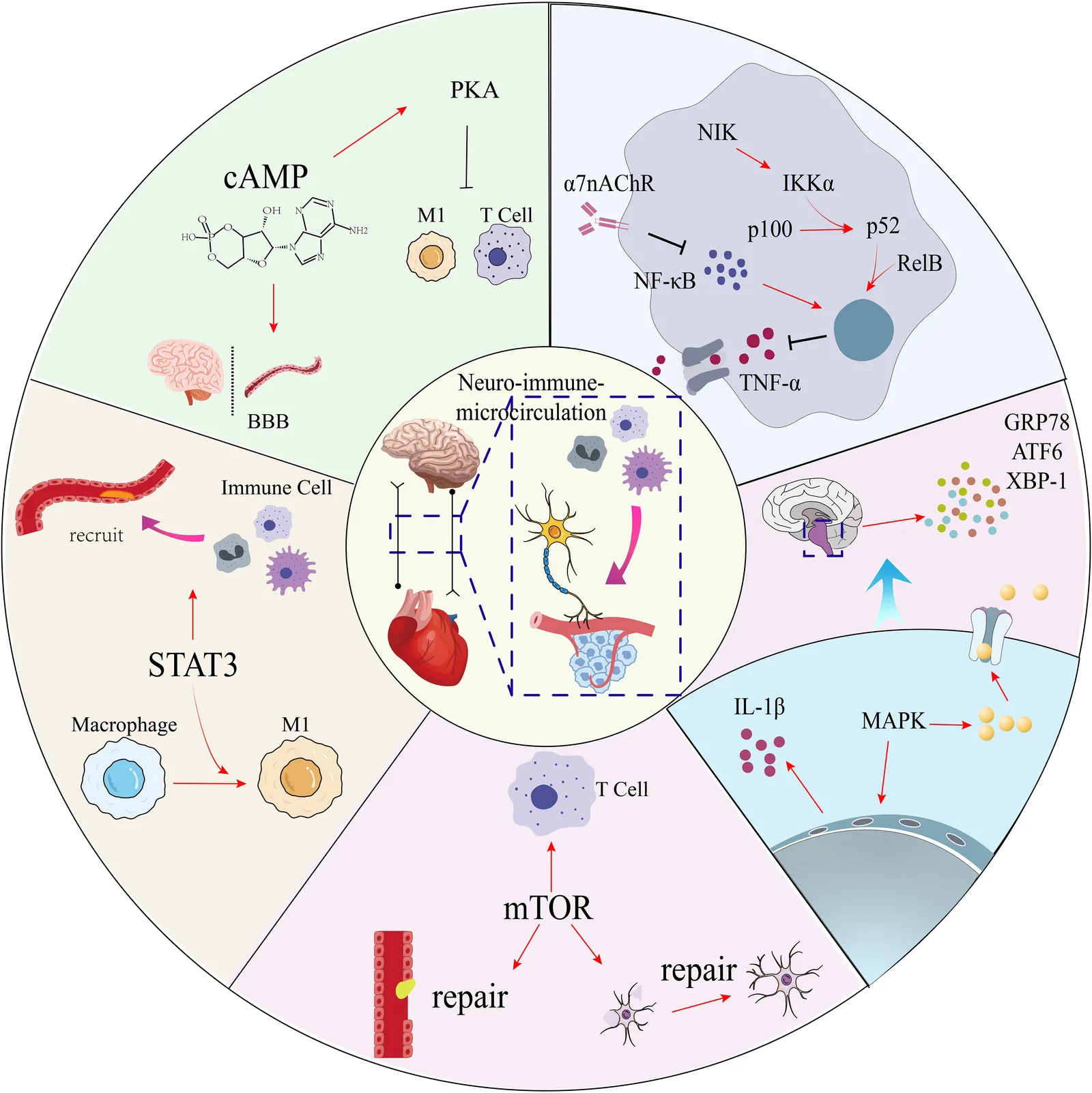
Neuroimmune signaling pathways in coronary microvascular disease. This diagram illustrates the balance between pro-inflammatory and protective intracellular pathways that regulate the central neuro-immune-microcirculatory axis. Pro-inflammatory signaling is driven by STAT3, which promotes M1 macrophage polarization, and by the MAPK pathway, activated by IL-1β. Cellular stress is mediated by the unfolded protein response (UPR). These are counter-regulated by protective mechanisms: the cholinergic pathway (via α7nAChR) inhibits NF-κB and TNF-α; cAMP/PKA signaling protects the blood-brain barrier (BBB) and dampens inflammation; and mTOR signaling promotes vascular and neural repair. STAT3, signal transducer and activator of transcription 3; MAPK, mitogen-activated protein kinase; IL-1β, interleukin-1 beta; UPR, unfolded protein response; α7nAChR, alpha-7 nicotinic acetylcholine receptor; NF-κB, nuclear factor kappa-light-chain-enhancer of activated B cells; TNF-α, tumor necrosis factor-alpha; cAMP, cyclic adenosine monophosphate; PKA, protein kinase A; BBB, blood-brain barrier; mTOR, mammalian target of rapamycin.

The cyclic AMP (cAMP) signaling pathway is a key homeostatic regulator within the neuro-immune-vascular axis, primarily exerting anti-inflammatory and barrier-stabilizing effects. Insights from research on the blood-brain barrier (BBB)—a precision microvascular model relevant for understanding general endothelial biology—demonstrate that increased intracellular cAMP enhances endothelial barrier function by regulating tight junctions and reduces neuroinflammation by suppressing the release of inflammatory mediators (104). This pathway is a direct target of the autonomic nervous system, as stimulation with beta-adrenergic receptor agonists like isoproterenol robustly increases cAMP levels (105). However, the functional outcome of cAMP signaling is highly context-dependent. While it stabilizes the barrier in macrovascular endothelium, studies on **coronary microvascular endothelial cells** have revealed that the same cAMP-elevating stimuli can paradoxically *increase* permeability. This is because the destabilizing effect of cAMP on cell adhesion structures (e.g., VE-cadherin) can override its beneficial, anti-contractile actions in this specific cell type (105). Furthermore, the efficacy of this entire pathway is tightly controlled by phosphodiesterases (PDEs), enzymes that degrade cAMP; their over-activity can lead to a state of cAMP deficiency and barrier dysfunction (104). These findings suggest a complex role for cAMP in CMVD, where its signaling may be both dysfunctional and contextually detrimental. We hypothesize that in the CMVD microenvironment, chronic sympathetic over-activity and local inflammation lead to a state of **compromised cAMP signaling**. This could manifest as either a blunted protective response due to receptor desensitization or increased PDE activity, or a paradoxical, barrier-disrupting response as observed in coronary endothelial cells, ultimately contributing to the persistent vasospasm, inflammation, and permeability characteristic of the disease. A literature analysis for these key signaling pathways is provided in **Table 1**, while their coordinated mechanism mediating microvascular inflammation and repair is summarized in **Figure 4**.

**Table 1:** Characteristics of key studies reshaping the diagnostic paradigm for coronary microvascular dysfunction (CMVD).

The signal pathway	Model(s)	Disease(s) studied	Evidence level	Mechanism description (Neuro-immune focus for CMVD)	Reference
The NF-κB Pathway	IL-34 knockout mouse model of myocardial ischemia/reperfusion (IR); Human acute coronary syndrome (ACS) patient samples.	Myocardial Ischemia/Reperfusion Injury.	Level II & III: Human biomarkers & *in vivo* mammalian model	This paper provides a direct mechanism for NF-κB activation in a cardiac ischemic setting. It shows that the cytokine IL-34, which is upregulated after IR, activates both the canonical (p65) and non-canonical (p52/RelB) NF-κB pathways in macrophages. This NF-κB activation is shown to be the direct upstream driver of the chemokine CCL2, which in turn mediates further macrophage recruitment and polarization, exacerbating cardiac remodeling. This provides a specific, citable pathway for how NF-κB drives macrophage-mediated inflammation in ischemic heart disease.	(91)
	Human coronary and carotid artery atherosclerotic lesion samples.	Atherosclerosis, Myocardial Infarction, Chronic Inflammatory Diseases.	Level II: Human observational study	This study focuses on the non-canonical NF-κB pathway specifically within the microvasculature. It demonstrates that the initiating kinase, NIK, is highly expressed in the endothelial cells of microvessels within atherosclerotic plaques. This activation is strongly associated with increased immune cell infiltration, an unstable plaque phenotype, and myocardial infarction. Crucially, it shows that these NIK-positive endothelial cells also express the leukocyte-recruiting chemokine CXCL12. This provides direct human evidence for the role of non-canonical NF-κB in driving inflammation directly from the microvascular endothelium.	(92)
The MAPK Pathway	Ischemia-induced heart failure rat model (Sprague-Dawley rats).	Heart Failure (HF)	Level III	This study provides direct evidence for a central neuro-immune mechanism in a cardiovascular disease model. It demonstrates that in HF, MAPK signaling is activated in key brain autonomic regulatory centers (SFO and PVN). This central activation leads to increased endoplasmic reticulum stress, heightened central inflammation, and critically, an enhanced sympathetic outflow, measured by plasma norepinephrine. For CMVD, this provides a strong mechanistic basis for how central MAPK activation could drive autonomic dysfunction and neuroinflammation, impacting the coronary microvasculature.	(95)
	Review article summarizing findings from various cellular (*in vitro*) and animal (*in vivo*) studies.	Hyperglycemia-induced cardiovascular complications (Diabetic Cardiomyopathy, Endothelial Dysfunction, Atherosclerosis).	Level V	This review focuses on peripheral vascular and immune mechanisms. It details how hyperglycemia, a common risk factor for CMVD, activates PKC-MAPK pathways in cardiovascular tissues. This activation drives key pathological processes: 1) Oxidative Stress: via activation of NADPH oxidase. 2) Inflammation: Upregulation of pro-inflammatory cytokines through activation of NF-kapper B by JNK and p38-MAPK. 3) Endothelial Dysfunction: Reduced nitric oxide (NO) bioavailability. For CMVD, this explains the local microvascular mechanisms, where metabolic stress activates MAPK to induce inflammation and vascular cell damage.	(96)
	Review article summarizing recent findings (last 5 years) from various models.	Heart Failure, Atherosclerosis, Myocardial Ischemia-Reperfusion Injury, Cardiac Hypertrophy.	Level V	This review reinforces the central role of MAPK in cardiovascular inflammation. For atherosclerosis, it highlights that p38 MAPK is overexpressed in macrophage-rich areas and that both p38 and JNK promote foam cell formation. It also describes MAPK’s role in endothelial-mesenchymal transition (EndMT) and VSMC proliferation. The paper confirms that overactivation of ERK1/2 and JNK1/2 in cardiac fibroblasts promotes myocardial fibrosis. For CMVD, this article provides broad, current support for MAPK as a key mediator of the inflammatory and remodeling processes involving macrophages, endothelial cells, and fibroblasts within the vessel wall.	(94)
	Review article summarizing findings from genetically modified murine models and pharmacological inhibitor studies.	Cardiac Hypertrophy, Myocardial Infarction, Atherosclerosis, Vascular Restenosis.	Level V	This foundational review provides specific molecular details. It explicitly states that JNK2 and p38 MAPK activity are required for macrophage foam cell formation, a key immune event in atherosclerosis. It also details how ERK1/2, JNK1/2, and p38 MAPK are all required for vascular smooth muscle cell (VSMC) proliferation and neointima formation after vascular injury. For CMVD, this provides a detailed molecular basis for how MAPK signaling directly controls the behavior of key immune (macrophages) and structural (SMCs) cells involved in adverse vascular remodeling.	(93)
The mTOR Pathway	Review article summarizing findings from various *in vivo* and *in vitro* models.	Tissue Regeneration (Neurons, Muscles, Liver, Intestine).	Level V	This review details the dual role of mTOR in the nervous system. Activation of mTOR (e.g., by deleting PTEN/TSC1) strongly promotes axonal regeneration after injury. However, hyper-activation of mTOR in astrocytes contributes to glial scar formation, which inhibits neuronal repair. This establishes mTOR’s powerful but context-dependent role in the CNS, providing a basis for hypothesizing its involvement in neuro-immune responses.	(98)
	Review article focusing on metabolic stress models (Western diet-fed rodents, etc.).	Overnutrition, Obesity, Cardiorenal Metabolic Syndrome, Insulin Resistance.	Level V	This review links metabolic stress (overnutrition) directly to enhanced mTOR signaling in cardiovascular tissues. It highlights that mTOR promotes a pro-inflammatory M1 macrophage polarization and modulates T-cell differentiation (Th1, Th17, Treg), which is central to the maladaptive immune response in obesity-related CVD. It also mentions mTOR’s link to the sympathetic nervous system. This provides a strong bridge between CMVD risk factors and mTOR-driven vascular inflammation.	(97)
	Rat model of repetitive ischemia; Human coronary artery endothelial cells (*in vitro*).	Coronary Collateral Growth, Mitochondrial Oxidative Stress.	Level III & IV	This experimental study provides a crucial counterpoint. It shows that mitochondrial oxidative stress (a key CMVD stressor) activates AMPK, which in turn inhibits mTOR signaling. This mTOR inhibition was found to be the direct cause of failed endothelial tube formation (a proxy for angiogenesis/repair). This perfectly addresses the “inhibition vs. activation” duality, showing that inappropriate mTOR inhibition can also be pathological by impairing necessary vascular repair.	(100)
	Review article focusing on T2DM models.	Type 2 Diabetes Mellitus (T2DM), Heart Failure, Diabetic Cardiomyopathy.	Level V	This review establishes the pathological consequences of chronic mTOR activation in the context of T2DM, a major CMVD risk factor. It explains that sustained mTORC1 activity creates a negative feedback loop that suppresses insulin signaling (IRS-1). It connects mTOR to cardiac hypertrophy, ischemia, and fibrosis. This grounds the “over-activation” part of the argument in a highly relevant disease model for CMVD.	(99)
The STAT3 Pathway	Angiotensin II-infused mouse model; Rat cardiomyocytes (*in vitro*).	Cardiac Remodeling, Hypertrophy, Fibrosis.	Level III & IV	This experimental paper perfectly details a key pathological role for STAT3. It shows that a pro-remodeling stimulus (Ang II) causes a biphasic activation of STAT3 in cardiomyocytes. An early, direct activation leads to IL-6 production, which in turn causes a delayed, sustained STAT3 activation that drives the expression of genes involved in hypertrophy and fibrosis. This provides a strong, citable mechanism for how STAT3 drives adverse remodeling in a cardiovascular context.	(101)
	Review article summarizing findings from various models.	Hepatocellular Carcinoma (HCC), Immune Microenvironment.	Level V	Although the disease context is oncology, this review provides an excellent, detailed overview of STAT3’s fundamental role as a master regulator of the immune system. It highlights STAT3’s dual function: regulating the differentiation of various immune cells (T cells, B cells, MDSCs) while also fostering an immunosuppressive microenvironment in pathological states by restraining effector T cells and promoting regulatory T cells (Tregs). This establishes STAT3’s capability to orchestrate complex immune responses, a key aspect of vascular inflammation.	(102)
	Endothelial progenitor cells (EPCs) from coronary artery disease (CAD) patients; Mouse model of hindlimb ischemia.	Coronary Artery Disease (CAD), Angiogenesis, Endothelial Progenitor Cells.	Level II, III & IV	This is a crucial paper that provides the “duality” argument. It demonstrates a protective/reparative role for STAT3 in a highly relevant context (CAD). The study found that in EPCs, STAT3 activation is essential for driving VEGFA-mediated angiogenesis in response to ischemic/acidic signals. Impaired STAT3 activation in cells from CAD patients correlated with poor angiogenic capacity. This is direct evidence that proper STAT3 function is necessary for vascular repair, countering the narrative that it is purely pathological.	(103)
The cAMP Pathway	Coronary endothelial cells (CEC) from rat; Aortic endothelial cells (AEC) from pig.	Endothelial Barrier Function, Vasodilation.	Level IV	(Neuro-Link & Duality Pillar) This paper provides a direct neuro-hormonal link by using the beta-adrenergic receptor agonist isoproterenol to stimulate cAMP, mimicking a sympathetic nervous system input. Critically, it reveals a profound duality: while cAMP signaling stabilizes the barrier in macrovascular cells, the same stimulation in coronary microvascular endothelial cells paradoxically increases permeability. This is because cAMP’s effect on disintegrating cell adhesion structures (VE-cadherin, paxillin) overrules its beneficial effect on relaxing contractile machinery. This is a powerful, highly specific mechanism for CMVD.	(105)
	Review article summarizing findings on the blood-brain barrier (BBB).	Alzheimer’s Disease (AD), Endothelial Barrier Function, Neuroinflammation.	Level V	(Fundamental Neuro-Immune & “Pathway Failure” Pillar) This review establishes cAMP’s fundamental role in maintaining endothelial barrier integrity by regulating tight junctions. It explains that cAMP signaling reduces neuroinflammation by suppressing inflammatory mediators. Importantly, it details how this protective pathway can fail through the over-activity of phosphodiesterases (PDEs), which degrade cAMP. Explanation for using this model: The BBB is the most specialized microvascular endothelial barrier. The molecular mechanisms by which cAMP regulates its permeability and inflammation provide a strong, generalizable model for understanding similar processes in other microvascular beds, like the coronary circulation, under chronic neuro-inflammatory stress.	(104)

#### 5.2.2 Soluble factors in neuro-immune interaction

Neurotransmitters not only exert a significant role as signal carriers within the nervous system, but they are also a regulator within the immune system. In arterial microvascular disease, neurotransmitters such as norepinephrine, glutamate, and CGRP are able to modulate the activity of immune cells by interacting with their receptors. NE is able to activate release from sympathetic nerve endings, act on β2-adrenergic receptors in the immune system, and inhibit the proinflammatory response of macrophages. This mechanism allows the sympathetic nervous system to modulate both the magnitude and persistence of immune activation, which in turn contributes to the pathogenesis of microvascular arterial disorders. Glutamate, an excitatory neurotransmitter secreted by the central nervous system, is able to transmit signals between neurons and immune cells via its NMDA receptor, inducing M1-type polarization of macrophages and enhancing inflammatory responses, which in turn exacerbate microvascular damage. CGRP, a neurotransmitter and vasomodulatory factor released by the sensory nerves, is known to have a potent vasodilatory effect.

Immune factors such as TNF-α and IL-1β act on endothelial cells through their corresponding receptors, inducing a pro-inflammatory response in endothelial cells, which in turn promotes the onset and progression of microvascular lesions.IL-6 (interleukin-6), a classical pro-inflammatory cytokine, has been widely studied in arterial microvascular disease.IL-6 is not only secreted by immune cells, but also activates the pro-inflammatory pathways and exacerbate inflammatory responses. Targeting the IL-6 signaling pathway (e.g., with IL-6 receptor antagonists) has been shown to have promising potential in the alleviation of inflammatory diseases.HMGB1 is a DAMP protein that is a major factor in tissue damage and inflammation.HMGB1 activates immune cells and promotes the secretion of inflammatory factors by binding to Toll-like receptors (TLRs), and also participates in neuro-immune interactions by influencing neurotransmitter secretion. Targeting HMGB1 or its receptor may become an emerging therapeutic approach for managing microvascular diseases.

#### 5.2.3 Neuro-immune potential for coronary microvascular diseases

Both immune and sympathetic nervous pathways are integral to the development and progression of CMVD. They also interact with each other, and their mechanisms of action are crucial to the treatment of coronary microvascular disease. With the deepening of the understanding of the neuro-immune interactions in arterial microvascular disease, an expanding volume of scientific literature has concentrated on the evolution of novel therapeutic approaches based on neuro-immune modulation. Exploring the potential applications of neuro-immune interactions in pharmacological interventions, emerging therapies, and personalized medicine can help to tap its significant value in improving the prognosis of arterial microvascular disease.

Due to the heterogeneity of arterial microvascular diseases, the neuro-immune interaction status varies significantly from patient to patient. Therefore, a stratified diagnosis and treatment approach based on patients’ neuro-immune characteristics may be the key to achieving precision medicine. In addition, in terms of biomarker discovery and application, the features of inflammatory factors expression by analyzing single-cell RNA sequencing data may provide a basis for early diagnosis and stratified diagnosis and treatment of coronary microvascular diseases.

Regulation of microvascular function is heavily influenced by the autonomic nervous system, with aberrant activation contributing to the development of multiple microvascular diseases. Pharmacological interventions targeting the autonomic nervous system have shown significant therapeutic potential. For example, β-blockers can alleviate arterial microangiopathy by inhibiting sympathetic nerve activity, improving endothelial-dependent vasodilation, and reducing vascular tone, thereby enhancing microvascular function (106). Studies have shown that carvedilol, a third-generation β-blocker, not only lowers blood pressure, but also protects the microvascular structure by reducing oxidative stress and inflammation, possibly through mechanisms involving the AMPK-Akt-eNOS signaling pathway (107). α-blockers (e.g., doxazosin) reduce vasoconstriction by blocking α_1_-adrenergic receptors on vascular smooth muscle, thereby reducing microvascular resistance and improving perfusion. Experimental studies have shown that doxazosin enhances endothelium-dependent vasodilation and vascular reactivity, supporting its role in microvascular protection (108). Cholinergic drugs (e.g., galantamine) can increase vagal activity and activate the cholinergic anti-inflammatory pathway, thereby attenuating systemic inflammation and protecting vascular endothelium. Studies indicate that galantamine suppresses pro-inflammatory cytokines (e.g., TNF-α), reduces oxidative stress, and mitigates endothelial injury, supporting its potential protective effects against arterial microangiopathy (109, 110). In addition, key molecules in neuro-immune interactions have emerged as important targets for the treatment of microvascular disease. Among them, IL-1 inhibitors, such as anakinra, have potential efficacy in microangiopathy by blocking IL-1 signaling, reducing inflammatory responses and protecting endothelial function, and NF-κB inhibitors can reduce neuroinflammation and immune stimulation by targeting the NF-κB signaling pathway, showing protective effects in experimental microangiopathy models. As a key player in neural-immune interactions, CGRP contributes to the pathophysiology of microvascular disorders through its association with inflammatory processes and vascular dysfunction when overactivated. CGRP antagonists are currently showing efficacy in migraine treatment, and we speculate that they may be used in the future to alleviate abnormal vascular responses in arterial microvascular disease.

In terms of emerging therapies, neuromodulatory techniques are a class of treatments that have emerged in recent years and are able to directly modulate nervous system activity through external or internal means, thereby affecting immune responses and microvascular function. Studies have shown that electrical activation of the VNS enhances parasympathetic activity, inhibits pro-inflammatory signaling, and attenuates microvascular inflammatory responses and endothelial damage. Transcranial magnetic stimulation (TMS) is able to non-invasively modulate cortical activity, indirectly improve microvascular function by affecting the brain-immune axis, reduce neuroinflammation and improve perfusion. More interestingly, cellular therapies based on neuro-immune interactions provide a completely new way of thinking about arterial microangiopathy treatment. The specificity and therapeutic effect of cells may be enhanced by genetic engineering or *in vitro* modifications. For example, by engineering modified T cells, key molecules in the neural-immune interaction can be precisely targeted to reduce inflammatory responses and restore vascular function. Mesenchymal stem cells (MSCs) can exert immunomodulatory and vascular repair capabilities by suppressing immune activation and promoting vascular regeneration. Although stem cell therapy has not yet seen direct application in coronary microvascular disease, it has shown positive results in animal models of diabetic microvascular disease.

## 6 Clinical implications and future perspectives

### 6.1 Key pharmacological strategies and clinical trials

The role of inflammation as a fundamental process in the initiation, progression, and destabilization of atherosclerotic plaques is now unequivocally established. Key pathological mechanisms include the activation of the NLRP3 (NOD-, LRR- and pyrin domain-containing protein 3) inflammasome within myeloid cells, which promotes the release of pro-inflammatory cytokines such as interleukin-1β. This understanding has propelled the investigation of targeted anti-inflammatory agents as a therapeutic strategy to mitigate residual cardiovascular risk beyond conventional lipid-lowering and antiplatelet therapies. Colchicine, a drug with a long history of clinical use, exerts broad anti-inflammatory effects primarily through disruption of microtubule dynamics. This interference impairs neutrophil chemotaxis, mobilization, and recruitment, while also indirectly inhibiting the oligomerization of the NLRP3 inflammasome (111). Recently, two landmark randomized controlled trials (RCTs), LoDoCo2 and COLCOT, have established that low-dose colchicine (0.5 mg daily), as an adjunct to standard secondary prevention therapy, significantly reduces the risk of major adverse cardiovascular events (MACE) in patients with stable coronary disease and recent myocardial infarction (112, 113). The success of these trials lends robust support to the concept of inflammation as a residual risk in atherosclerotic cardiovascular disease (ASCVD) and has directly spurred its investigation in the context of CMVD. Several trials are currently underway, most notably the COLHEART-PRESERVED (NCT06217120) study, which specifically enrolls patients with heart failure with preserved ejection fraction (HFpEF) and concomitant CMVD. This trial aims to directly assess whether colchicine can improve the primary endpoint of coronary flow reserve (CFR), thereby providing direct physiological evidence for the reversal of CMVD through anti-inflammatory therapy (114).

Several randomized controlled trials evaluating conventional anti-anginal vasodilators have reported neutral outcomes, highlighting the limitations of applying therapies designed for epicardial disease to the microvasculature. For example, the NIRVANA (NCT01665508) trial, which investigated the third-generation beta-blocker nebivolol in women with CMD, did not demonstrate a significant improvement in the co-primary endpoints of angina frequency and quality of life as measured by the Seattle Angina Questionnaire (115). Similarly, the EDIT-CMD (NCT04777045) trial reported that the non-dihydropyridine calcium channel blocker diltiazem failed to improve its primary endpoint of coronary flow velocity reserve (116). However, a pre-specified secondary analysis did note a reduction in the prevalence of acetylcholine-provoked epicardial spasm, suggesting a potential niche for this agent in patients with a vasospastic component.

Investigations into other pharmacological pathways have also been met with challenges. The PRIZE (NCT04097314) trial tested the hypothesis that selective antagonism of the endothelin-A receptor with zibotentan could improve microvascular function (117). The rationale was based on the vasoconstrictive effects of endothelin-1. Despite this targeted approach, the trial was negative, as zibotentan did not improve the primary endpoint of treadmill exercise duration compared to placebo. The collective results of these trials underscore the complexity of CMD and suggest that a single pharmacological approach may be insufficient for this heterogeneous patient population.

In contrast to the outcomes with small-molecule drugs, novel biologic and regenerative therapies are emerging as a potential avenue. The ESCaPE-CMD (NCT03508609) trial was designed to evaluate the safety and potential bioactivity of a novel regenerative therapy, intracoronary administration of autologous CD34+ cells (CLBS16) (118). The proposed mechanism of action involves the promotion of microvascular repair and neovascularization. The study successfully met its primary safety endpoint. Regarding efficacy, the trial demonstrated a statistically significant improvement in coronary flow reserve (CFR)—an objective physiological marker—at the 6-month follow-up in the treatment group compared to the sham-controlled group. These findings provide a promising, albeit preliminary, signal supporting the therapeutic potential of this intervention.

Early research efforts have alluded to the potential benefits of targeting specific patient subgroups. For instance, the pilot study NCT00570089 investigated the late sodium current inhibitor ranolazine, not in a broad NOCAD population, but specifically in women with documented myocardial ischemia in the absence of obstructive disease (119). While limited by its small sample size, the study reported trends toward improvement in angina symptoms and quality of life, suggesting that mechanism-specific drugs may hold value for select patient profiles.

The core tenets of precision medicine are most explicitly manifested in modern, mechanism-driven clinical trial designs, which tailor interventions based on underlying pathophysiological processes. The ongoing EXAMINE-CAD (NCT05294887) trial serves as a prime example of this advanced approach. The study protocol mandates initial invasive coronary function testing to phenotype patients and stratify them into endotypes, such as those with predominantly microvascular dysfunction versus those with a significant vasospastic component (120). Following stratification, the trial employs a randomized, crossover design to directly compare the therapeutic efficacy of two agents with distinct mechanisms of action: the beta-1 selective blocker bisoprolol and the non-dihydropyridine calcium channel blocker diltiazem.

The fundamental objective of trials like EXAMINE-CAD is to move beyond the question of if a drug works in a broad population, to in which specific patient phenotype a drug provides optimal benefit. The outcomes of such mechanism-based studies are highly anticipated, as they hold the potential to provide high-level evidence to inform the development of personalized treatment algorithms. By tailoring pharmacotherapy to the dominant underlying pathophysiology, this approach aims to improve clinical outcomes for the large and challenging population of patients with symptomatic NOCAD. The design, key characteristics, and primary outcomes of the clinical trials discussed in this review are summarized in **Table 2**.

**Table 2:** Key trials shaping the therapeutic landscape for coronary microvascular dysfunction (CMVD).

Drug name	Target/Mechanism of action	Disease Treated	Trial Name/Alias	Phase	Registration no.	Sample size	Summary of key results	Reference
Ranolazine	Inhibition of late inward sodium current (late I_Na_), reducing intracellular calcium overload.	Microvascular Coronary Disease/Angina	Microvascular Coronary Disease In Women: Impact Of Ranolazine	Phase 2	NCT00570089	20	Ranolazine improved angina, as measured by the Seattle Angina Questionnaire, and showed a trend toward improved myocardial perfusion.	(119)
Zibotentan	Selective Endothelin-A (ET-A) Receptor Antagonist	Microvascular Angina (MVA)	PRIZE	Phase 2a	NCT04097314	118	Failed to improve exercise tolerance and had more adverse events than placebo.	(117)
Nebivolol	Highly selective beta-1 adrenergic receptor antagonist with additional nitric oxide (NO)-mediated vasodilatory effects.	Microvascular Angina/Coronary Microvascular Dysfunction	NIRVANA	Phase 4	NCT01665508	35	Trial did not meet its primary endpoint. Nebivolol did not significantly improve angina frequency or quality of life (measured by Seattle Angina Questionnaire) compared to placebo.	(115)
Colchicine (Low-Dose)	Anti-inflammatory; inhibits neutrophil activity and the NLRP3 inflammasome, reducing the inflammatory response in atherosclerosis	Chronic Coronary Disease (for secondary prevention)	LoDoCo2	Phase 3	ACTRN12614000093684	5,522	Low-dose colchicine significantly reduced the primary composite endpoint of cardiovascular death, myocardial infarction, ischemic stroke, or ischemia-driven revascularization compared to placebo.	(112)
		Secondary prevention after recent Myocardial Infarction (MI)	COLCOT	Phase 3	NCT02551094	4,745	Compared to placebo, colchicine significantly reduced the risk of the primary composite endpoint (cardiovascular death, MI, stroke, resuscitated cardiac arrest, or urgent hospitalization for angina requiring revascularization) in patients with a recent MI.	(113)
		Heart Failure with Preserved Ejection Fraction (HFpEF) and Coronary Microvascular Dysfunction (CMD)	COL-Micro-HF	Phase 2	NCT06217120	50	The study is currently active and recruiting participants. Primary results are not yet available.	(114)
Diltiazem	Non-dihydropyridine calcium channel blocker (CCB); inhibits calcium influx into cardiac and vascular smooth muscle, causing coronary and systemic vasodilation.	Coronary Microvascular Dysfunction (CMD)/Angina with Non-Obstructive Coronary Arteries (ANOCA)	EDIT-CMD	Phase 4	NCT04777045	85	Trial did not meet its primary endpoint. Diltiazem did not substantially improve coronary vasomotor dysfunction, symptoms, or quality of life compared to placebo. However, it did reduce the prevalence of epicardial spasm.	(116)
Bisoprolol/Diltiazem	Beta-Blocker/Calcium Channel Blocker	Coronary Microvascular Dysfunction (CMD), INOCA	EXAMINE-CAD	Phase 2	NCT05294887	132	The study is currently active and recruiting participants. Primary results are not yet available.	(120)
CLBS16 (Autologous CD34+ Cells)	Pro-angiogenic cell therapy; promotes repair and regeneration of the microvasculature through neovascularization.	Coronary Microvascular Dysfunction (CMD)	ESCaPE-CMD	Phase 2a	NCT03508609	20	The study met its primary safety endpoint. Intracoronary delivery of CLBS16 was well-tolerated. It also showed a statistically significant improvement in coronary flow reserve (CFR) at 6 months compared to a sham-treated control group.	(118)

### 6.2 Emerging diagnostic tools for inflammatory phenotyping in CMVD

The PROMIS-HFpEF study, a landmark prospective, multinational, multicenter observational investigation, provided pivotal clinical evidence elucidating the systemic nature of CMVD (121). In this study, coronary flow reserve (CFR) was noninvasively assessed in rigorously characterized patients with heart failure with preserved ejection fraction (HFpEF) using transthoracic Doppler echocardiography under adenosine-induced hyperemia. Concurrently, systemic endothelial function was evaluated via peripheral arterial tonometry.

The study revealed a remarkably high prevalence of CMVD—defined as CFR < 2.5—in up to 75% of HFpEF patients. More importantly, it demonstrated, for the first time, a significant and independent association between impaired CFR and markers of systemic endothelial injury, including elevated urinary albumin-to-creatinine ratio (UACR) and reduced reactive hyperemia index (RHI). These findings challenge the conventional view of CMVD as a cardiac-isolated phenomenon and instead suggest that CMVD may represent a localized cardiac manifestation of broader systemic inflammatory and endothelial dysregulation. This paradigm shift provides a robust theoretical basis for investigating CMVD pathogenesis from neuroimmune and systemic perspectives.

Following the recognition of CMVD as a systemic disorder, dissecting its functional heterogeneity at the individual level necessitates clinical validation using invasive “gold standard” techniques. Two pivotal randomized controlled trials—CorCTCA (NCT03477890) and iCorMicA (NCT04674449)—have been specifically designed to address this objective. Both studies enrolled patients with angina and no obstructive coronary artery disease (INOCA), implementing a comprehensive invasive functional assessment protocol. This protocol includes guidewire-based measurements of coronary flow reserve (CFR) and index of microcirculatory resistance (IMR), as well as acetylcholine (ACh) provocation testing to evaluate endothelium-dependent and vasospastic dysfunction (122, 123).

The completed CorCTCA trial demonstrated considerable success at the diagnostic level, clearly stratifying patients into distinct pathophysiological endotypes—namely microvascular, vasospastic, or mixed forms of dysfunction. However, a paradoxical insight emerged from the 12-month follow-up: enhanced diagnostic precision did not translate into significant symptomatic improvement in angina. This “negative” therapeutic outcome constitutes perhaps the most thought-provoking aspect of the study, as it exposes a critical gap between diagnostic capability and effective treatment strategies in current clinical practice.

Notably, the use of acetylcholine, a key neurotransmitter, directly connects invasive diagnostics with neurovascular regulatory function, further underscoring the neuroimmune dimensions of CMVD pathophysiology. Against this backdrop, the ongoing iCorMicA trial carries heightened expectations. It aims to determine whether personalized, stratified therapeutic approaches—guided by precise endotype-based diagnosis—can effectively bridge the current “diagnosis-treatment gap” and deliver meaningful clinical benefit to patients.

Although invasive testing remains the current “gold standard” for assessing CMVD, its inherent limitations—including procedural invasiveness, high cost, and poor reproducibility—have constrained its widespread implementation in clinical practice. As a result, the development of reliable noninvasive diagnostic tools has become an inevitable trend in the field. The Imaging CMD Study (NCT05634031) exemplifies this direction, aiming to establish and validate a PET-based noninvasive diagnostic protocol centered on myocardial perfusion imaging, with direct comparisons to invasive physiological assessments (124). However, the broader significance of this study extends well beyond its immediate diagnostic objective. It represents a critical step toward the future of integrated, multimodal molecular imaging in the assessment of CMVD. The true potential of PET imaging lies in its versatility—not only enabling the visualization of myocardial blood flow, but also allowing for the interrogation of underlying molecular and cellular processes through the use of targeted radiotracers. Looking ahead, a PET-based diagnostic platform may allow for simultaneous, quantitative, and spatially resolved assessment of myocardial microvascular function, local inflammation, and autonomic innervation in a single imaging session. This could be achieved by combining ^13N-ammonia (for perfusion), ^18F-FDG (for inflammation), and ^11C-HED (for sympathetic innervation). Such an approach would enable, for the first time *in vivo*, a direct visualization of the dynamic interplay among perfusion abnormalities, inflammatory activity, and neurovascular dysregulation in CMVD—marking a paradigm shift from indirect inference to direct observation in cardiovascular research. The core design and characteristics of these key trials are summarized in **Table 3**.

**Table 3:** Characteristics of key studies reshaping the diagnostic paradigm for coronary microvascular dysfunction (CMVD).

Study name	Study type	Registration number	Sample size	Target disease	Diagnostic techniques/Methods	Primary endpoints/Main outcome measures	Results	Reference
PROMIS-HFpEF	Observational Study	N/A	202 HFpEF patients with successful Coronary Flow Reserve (CFR) testing	Heart Failure with preserved Ejection Fraction (HFpEF) & Coronary Microvascular Dysfunction (CMD)	Coronary Flow Reserve (CFR) ;Measurement, Systemic Endothelial Function Measurement	1. Prevalence of CMD (defined as CFR < 2.5) in the HFpEF population. 2. Correlation of CMD with systemic endothelial dysfunction, clinical factors, lab markers, and echocardiographic indices.	1. A high prevalence of CMD (75%) was found in patients with HFpEF. 2. Worse CFR was significantly associated with higher NT-proBNP, higher urinary albumin-to-creatinine ratio (UACR), lower RHI, and right ventricular (RV) dysfunction. 3. History of smoking and atrial fibrillation were the comorbidities most closely associated with CMD.	(121)
CorCTCA	Clinical Trial	NCT03477890	250 patients underwent invasive procedure; 231 were randomized	Angina with Non-Obstructive Coronary Arteries (ANOCA), including microvascular and vasospastic angina	Initial Screen: CT Coronary Angiography (CTCA). Core Diagnostic: Invasive coronary function assessment, including Fractional Flow Reserve (FFR), Coronary Flow Reserve (CFR), Index of Microvascular Resistance (IMR), and acetylcholine (ACh) provocation testing.	1. Diagnostic Endpoint: Reclassification rate of the initial CTCA diagnosis based on invasive functional assessment. 2. Randomized Trial Endpoint: Change from baseline in the Seattle Angina Questionnaire (SAQ) score at 6 months.	1. Diagnostic Reclassification: Invasive assessment specified diagnoses in non-obstructive CAD patients: 55.0% had microvascular angina, 11.7% vasospastic angina, and 7.4% had both. The diagnosis of a coronary vasomotor disorder was 4-fold more likely in the intervention group versus control. 2. Angina Improvement: No significant difference in SAQ score improvement was observed between the intervention and control groups at 6 or 12 months. 3. Other Findings: Despite no significant improvement in angina, treatment satisfaction was significantly higher in the intervention group at 12 months.	(122)
iCorMicA	Clinical Trial	NCT04674449	400 participants	Ischemia with Non-Obstructive Coronary Arteries (INOCA), including microvascular and vasospastic angina	Invasive coronary function tests, including Fractional Flow Reserve (FFR), Coronary Flow Reserve (CFR), Index of Microcirculatory Resistance (IMR), and acetylcholine (ACh) provocation testing.	Change from baseline in Seattle Angina Questionnaire (SAQ) quality of life score at 6 months.	The trial is currently recruiting. The estimated primary completion date is October 2026, therefore no final results are available yet.	(123)
IMAGING-CMD	Clinical Trial	NCT05634031	70 participants	Coronary Microvascular Dysfunction (CMD); Ischemia with Non-Obstructive Coronary Arteries (INOCA)	Core Non-invasive: Cardiac PET Myocardial Perfusion Imaging (PET MPI). Invasive Validation (sub-study): For a subset with abnormal PET, invasive functional angiography (FFR, CFR, IMR, ACh testing) will be performed.	1. To validate the PET-based diagnostic algorithm against invasive functional assessment. 2. To assess improvement in symptoms, function, and quality of life following PET-guided management.	The trial is currently recruiting. The estimated primary completion date is December 2026, therefore no final results are available yet.	(124)

In summary, this series of logically progressive clinical trials has collectively established a novel framework for the diagnosis and investigation of CMVD, while providing critical insights for future exploration of neuroimmune mechanisms. First, forthcoming clinical studies must move beyond the outdated notion of CMVD as a homogeneous disease entity and instead utilize validated invasive or emerging non-invasive phenotyping tools to enable precise patient stratification and enrollment. Such approach will facilitate the assessment of therapeutic efficacy within populations defined by shared pathophysiological profiles. Second, endpoint measures in trials targeting neuroimmune pathways must be correspondingly refined. These should incorporate a composite of symptom improvement, objective functional indices—such as coronary flow reserve (CFR) and index of microvascular resistance (IMR)—and molecular imaging biomarkers capable of directly reflecting target engagement (e.g., PET-quantified myocardial inflammation or alterations in cardiac neural density). By integrating these advanced diagnostic strategies, the field of CMVD is now poised at a historical turning point—shifting from largely descriptive association studies to a new era of mechanism-driven, precision diagnosis-based clinical trials. Ultimately, this will address the central scientific question: can modulation of the neuroimmune axis reverse CMVD pathophysiology and improve patient outcomes?

## 7 Challenges and future research directions

Recent technological advancements in genetics and artificial intelligence have enabled precise modulation of neurons in type- and region-specific manner. These technologies now allow for the purpose of targeted excitation or inhibition of selected subpopulations of neurons in discrete regions of the nervous system, through genetic methods of inserting activating ion channels into individual neurons or the engineering drug-selectively activated designer receptors (86). A genetics–based investigation demonstrated that various areas of the brain differentially and swiftly regulate immune cell dynamics in response to stress, which may compromise immune defenses and elevate the risk of disease (87). At the same time, these findings highlight the potential of targeted immunomodulation for cardiovascular disease. The emergence of genetic tools—including optogenetics, chemical genetics and genome editing—offers powerful approaches to selectively modulate the neuroimmune axis implicated in cardiovascular disease critical for realizing tissue-targeted immunomodulation with no guarantee of generalized immunosuppression or other non-targeted effects, and hold significant promise for identifying novel therapeutic targets for precision medicine.

In essence, our current understanding of the interactions among the nervous, cardiovascular, and immune systems make contributions toward prevention and management of cardiovascular disease. However, further comprehensive and mechanistic research are needed to elucidate how peripheral inflammation stimulates the neuronal pathways, and to clarify how the nervous and stress responses modulate immune cell function at sites of inflammation. Advancing this knowledge will not only deepen our understanding of cardiovascular disease pathophysiology, but also support the optimization of existing therapies and the development of more effective, targeted treatment strategies to improve patient outcomes.

## Equations

The equations should be inserted in editable format from the equation editor.


$$\text{f}\left(\text{x}\right){\text{=a}}_{0}+\sum_{n=1}^{\infty }\left({\text{a}}_{\text{n}}\text{cos}\frac{\text{nπx}}{\text{L}}{\text{+b}}_{\text{n}}\text{sin}\frac{\text{nπx}}{\text{L}}\right)$$
